# Physico-Chemical and Biological Evaluation of Spin-Coated Chromium-Doped Hydroxyapatite in Dextran Matrix Coatings

**DOI:** 10.3390/biomimetics11050327

**Published:** 2026-05-07

**Authors:** Simona Liliana Iconaru, Steluta Carmen Ciobanu, Coralia Bleotu, Mikael Motelica-Heino, Daniela Predoi

**Affiliations:** 1 National Institute of Materials Physics, Atomistilor Street, No. 405A, P.O. Box MG 07, 077125 Magurele, Romania; simonaiconaru@gmail.com; 2 Department of Cellular and Molecular Pathology, Stefan S. Nicolau Institute of Virology, 030304 Bucharest, Romania; cbleotu@yahoo.com; 3 Research Institute of the University of Bucharest (ICUB), University of Bucharest, 060023 Bucharest, Romania; 4 The Academy of Romanian Scientist, 050711 Bucharest, Romania; 5 Department of Civil Engineering and Environment, Institut des Sciences de la Terre d’Orléans (ISTO), Université d’Orléans, UMR 7327 CNRS, 1A Rue de la Férollerie, 45071 Orléans, France; mikael.motelica@univ-orleans.fr

**Keywords:** spin-coated films, antimicrobial activity, biocompatibility, Cr-substituted hydroxyapatite, dextran matrix

## Abstract

This study reports on the physico-chemical and in vitro biological characterization of chromium-doped hydroxyapatite (10CrHAp, Cr^3+^, Ca_10-x_Cr_x_(PO_4_)_6_(OH)_2_, x_Cr_ = 0.1) and chromium-doped hydroxyapatite in dextran matrix (10CrHAp-Dx) coatings, prepared for the first time via the spin coating technique. X-ray diffraction analysis and Rietveld refinement were used to characterize the materials. Fourier-transform infrared (FTIR) spectroscopy confirmed the presence of functional groups specific to hydroxyapatite. Scanning electron microscopy (SEM) observations revealed the presence of a conglomerate of nanoparticles distributed unevenly across the coatings surface. Atomic force microscopy (AFM) showed that both coatings presented continuous surfaces with uniform morphology. The in vitro biocompatibility of 10CrHAp and 10CrHAp-Dx coatings was evaluated using human osteoblast-like MG63 cell line and MTT assay. SEM and MM visualization assessed the cell adhesion and proliferation and morphological changes in the adhered cells. The antibacterial properties of the 10CrHAp and 10CrHAp-Dx coatings was assessed in vitro against two of the most common bacterial reference strains, *Pseudomonas aeruginosa* ATCC 27853 and *Staphylococcus aureus* ATCC 25923. Overall, the coatings achieved log reductions up to ~9.35, corresponding to a bacterial kill rate (for *S. aureus*) exceeding 99.99%, with 10CrHAp-Dx showing slightly superior performance. Similar behavior (log reductions of ~8.6 and ~8.9, respectively, indicating a sustained antibacterial effect and >99.99% bacterial elimination) was observed and for *Pseudomonas aeruginosa*. AFM was used to evaluate the bacterial cells interaction with the coating’s surfaces. The biological assays demonstrated that both coatings possess notable antibacterial activity, underscoring their potential in biomedical applications, particularly in the design of new antimicrobial devices.

## 1. Introduction

Hydroxyapatite (HAp; Ca_10_(PO_4_)_6_(OH)_2_) is one of the most extensively investigated materials within the field of biomaterials. HAp is the principal inorganic component of bone and teeth and remains an important material in hard-tissue engineering due to its chemical similarity to biological apatite, inherent osteoconductivity, and general biocompatibility. However, the stoichiometric HAp ceramics are brittle and typically lack intrinsic antimicrobial function, which limits their performance as implant coatings in infection-prone environments. In order to be able to overcome these limitations, researchers commonly substitute biologically relevant or therapeutic ions into the HAp lattice, and combine HAp with polymers to form composite coatings that improve toughness, adhesion to metallic substrates, and provide functional interfaces for drug/ion release. The physicochemical and biological properties of HAp can be improved through controlled modifications [1,2,3,4]. The crystalline lattice of HAp comprises two distinct calcium sites (Ca^2+^), which provide substitutional flexibility and thereby exert a pronounced influence on its physicochemical behavior [5]. Considerable research has examined the effects of doping HAp with divalent cations, including Mg^2+^ and Zn^2+^ [4,5,6,7,8]. Furthermore, the incorporation of trivalent ions such as Cr^3+^ and Eu^3+^ has been explored as a means of improving various properties [9,10,11,12,13,14]. Chromium, although being known as an essential trace element in the human body, exhibits a concentration-dependent toxicity [12,13]. Cr (III) is generally considered less cytotoxic than Cr(VI), attributable to its lower solubility and limited cellular uptake [15]. Hydroxyapatite, due to its superior bioactivity and osteoconductivity, continues to be widely employed in bone tissue engineering [16,17,18,19]. Chromium (especially trivalent Cr^3+^) has been explored as dopant for hydroxyapatite in order to enhance its properties. Cr^3+^ ions can substitute into the calcium sites in HAp, potentially altering crystallinity and morphology, and introducing antibacterial or catalytic functionalities, while in certain oxidation states and concentrations maintaining biocompatibility. However, careful control of chromium speciation and concentration is essential, since higher oxidation states (e.g., Cr^6+^) are toxic [19,20,21].

Therefore, in recent years, several studies reported that embedding HAp and doped HAp in a biopolymeric matrix is a widely used strategy in order to improve adhesion to metal substrates, to reduce brittleness, and also to enable a sustained release of some therapeutic agents or ions [7,19,20,21,22,23,24,25,26,27]. Polysaccharide coatings or matrices such as dextran, which is a hydrophilic, biocompatible polysaccharide, offer several advantages when combined with HAp. They can improve particle dispersion, provide biocompatible interfaces, act as stabilizers, and can help in drug loading or controlled release [28,29]. Dextran is particularly attractive because of its biodegradability, non-toxicity, hydrophilicity, and well-known use in biomedical contexts (e.g., as stabilizer, carrier, etc.). Several studies (including dextran-coated Zn- or Mg-doped HAp and dextran-enriched HAp thin films) demonstrate that dextran matrices improve dispersion and film homogeneity, and can be combined with dip-coating processes to obtain uniform composite layers with promising in vitro bioactivity and controlled degradation behavior. These findings support the use of dextran as a matrix for 10CrHAp coatings to enhance the mineral bioactivity of HAp [29,30,31,32,33]. Therefore, the combination of chromium-doped hydroxyapatite particles coated by a dextran matrix, and applied as coatings on substrates (e.g., implants or devices), represents a promising strategy to develop multifunctional biomaterials. Such a composite coating could combine good mechanical adhesion, osteoconduction, and biocompatibility, enhanced antibacterial behavior, and possibly controlled release of therapeutic agents or ions [29,30,31,32,33].

In this work, we focus on the synthesis, structural and chemical characterization, and in vitro evaluation of chromium-doped hydroxyapatite and chromium-doped hydroxyapatite in dextran matrix coatings. Thus, novelty of this manuscript mainly consists of the development for the first time of 10CrHAp-based coatings (Cr^3+^, x_Cr_ = 0.1, 10CrHAp; 10CrHAp-Dx) by the spin coating method. More than that, various techniques were employed to study the obtained coatings, such as X-ray diffraction (XRD), X-ray photoelectron spectroscopy (XPS), Fourier-transform infrared (FTIR) spectroscopy, scanning electron microscopy (SEM), X-ray energy-dispersive spectroscopy (EDX), atomic force microscopy (AFM) and metallographic microscopy (MM). Furthermore, the biocompatibility and antimicrobial activity of the new developed coatings were evaluated for the first time.

## 2. Materials and Methods

### 2.1. Materials

Chrome-doped hydroxyapatite (Ca_10-x_Cr_x_(PO_4_)_6_(OH)_2_, Cr^3+^, 10CrHAp, x_Cr_ = 0.1; [Ca + Cr]/P = 1.67) and chrome-doped hydroxyapatite in dextran matrix (Cr^3+^, 10CrHAp-Dx, x_Cr_ = 0.1; [Ca + Cr]/P = 1.67) were synthesized by an adapted sol–gel method. The precursors used in the synthesis process included calcium nitrate (Ca(NO_3_)_2_·4H_2_O, Sigma-Aldrich, St. Louis, MA, USA), chromium nitrate (Cr(NO_3_)_3_·9H_2_O, Alfa Aesar, Karlsruhe, Germany), ammonium hydrogen phosphate ((NH_4_)_2_HPO_4_, Alfa Aesar, Karlsruhe, Germany), dextran (H(C_6_H_10_O_5_)n, MW ∼ 40.000, Sigma-Aldrich, St. Louis, MO, USA), and ethanol (Sigma-Aldrich, St. Louis, MO, USA). The 10CrHAp and 10CrHAp-Dx coatings were deposited onto silicon (Si) substrates (Siegert Wafer GmbH, Aachen, Germany) using the spin coating technique.

### 2.2. Synthesis of Chrome-Doped Hydroxyapatite and Chrome-Doped Hydroxyapatite in Dextran Matrix Coatings

The synthesis of 10CrHAp involved the preparation of two separate ethanol-based solutions: the first solution consisted of a phosphate solution (0.5 mol/L solution) and the second solution (1.67 mol/L) consisted of a calcium and chromium nitrate solution. Both solutions were stirred at 40 °C for 2 h. Then, the phosphate solution was gradually added to the calcium and chromium nitrate solution under continuous and vigorous stirring, followed by 12 h of reaction at 80 °C while maintaining a pH of 10. These steps were made under ambient conditions. The resulting gel was washed multiple times (five times) with deionized water and ethanol, redispersed in ethanol, and stirred for an additional 12 h. On the other hand, for the synthesis of 10CrHAp-Dx followed the same process as the one previously described. In this case, after the final wash, the obtained material was added in a 10% dextran solution and subsequently stirred for 12 h at 60 °C under ambient air conditions. The detailed procedure was presented in the study reported by Ciobanu C.S. et al. [19].

The final solutions (10CrHAp and 10CrHAp-Dx) were used for the deposition of 10CrHAp and 10CrHAp-Dx coatings onto Si substrates via spin coating. The Si disks used for the coating’s deposition was ultrasonically cleaned and rinsed with acetone and distilled water prior to deposition. Then 0.5 mL of the final solution dispensed onto the Si substrate with the aid of a syringe. The parameters used for the spin coating procedure were: a speed of 2000 rpm, spin time of 60 s and acceleration ramp of 800 rpm/s. The deposition step was repeated 30 times (under ambient conditions) in order to achieve the 10CrHAp and 10CrHAp-Dx coatings. After the last deposition, the 10CrHAp and 10CrHAp-Dx coatings were subjected to a thermal treatment at 80 °C for 72 h in air.

### 2.3. Methods

The structural analysis of 10CrHAp and 10CrHAp-Dx samples was performed using a Bruker D8 Advance X-ray diffractometer (Bruker, Karlsruhe, Germany) equipped with CuKα radiation (λ = 1.5418 Å) and a LynxEye™ detector, ensuring high resolution and detection efficiency. The XRD data were collected over a 15–75° 2θ range with a step size of 0.02°, allowing for the precise identification of crystalline phases and peak positions. The structural parameters were obtained following the procedure previously described by Ciobanu et al. [27]. Rietveld refinement of the XRD patterns for the 10CrHAp and 10CrHAp Dx samples was performed using MAUD software (version 2.9995) [34]. A fifth-order polynomial modeled the background, and peak profiles were fitted with a pseudo-Voigt function. Refinement employed a least-square weighting scheme and proceeded until the calculated patterns closely matched the experimental data.

The 10CrHAp and 10CrHAp-Dx were also analyzed using X-ray Photoelectron Spectroscopy (XPS) technique. Surface chemical analysis was performed using a SES 2002 spectrometer (Scienta Omicron, Taunusstein, Germany) equipped with a monochromatic Al Kα X-ray source (1486.6 eV). The obtained XPS data were studied using CasaXPS version 2.3.14, applying Shirley background subtraction [33,35]. Charge correction was uniformly applied using the C 1s peak at 284.8 eV as the binding energy reference.

Fourier-transform infrared (FTIR) spectroscopy was used to analyze the vibrational modes of the functional groups present in 10CrHAp and 10CrHAp-Dx coatings. The measurements were carried out using a Perkin Elmer spectrometer (Waltham, MA, USA). equipped with an attenuated total reflection (ATR) accessory. FTIR spectra were recorded within the 450–3800 cm^−1^ range under ambient conditions. Each spectrum was obtained by using the next parameters: 32 scans at a spectral resolution of 4 cm^−1^. Additionally, both second-derivative spectra (obtained for the 450–1500 cm^−1^ range) and deconvoluted spectra were obtained for the 400–700 cm^−1^, 800–1200 cm^−1^, and 1350–1700 cm^−1^ spectral regions for the 10CrHAp and 10CrHAp-Dx coatings [36]. These steps were performed since second-derivative FTIR spectra improve spectral resolution by revealing hidden or overlapping bands, thus enabling precise identification of functional group vibrations. On the other hand, FTIR deconvoluted spectra further separates overlapping absorption bands, providing detailed insight into the chemical structure and crystallinity of the material.

Data about the morphological characteristics of the 10CrHAp and 10CrHAp-Dx coatings were obtained using scanning electron microscopy (SEM) and metallographic microscopy (MM). SEM observations were performed with a Hitachi S-4500 (Hitachi, Tokyo, Japan) scanning electron microscope. Moreover, preliminary information regarding the coatings’ thickness was obtained by performing transversal cross-section SEM studies. Complementary information on the surface morphology of the 10CrHAp and 10CrHAp-Dx samples was obtained through MM using an inverted trinocular metallographic microscope (OX.2153-PLM, Euromex, Arnhem, The Netherlands). The MM images were obtained using a 50× magnification objective. The SEM and MM images were further analyzed with ImageJ software (version 1.51j8), which was also used to obtain the 3D surface profiles from the metallographic images [37].

The adhesion features of 10CrHAp and 10CrHAp-Dx coatings were evaluated using an adhesive tape pull test, using 3M Performance Flatback 2525 adhesive tape (peel adhesion was 7.5 N/cm).

Information regarding the surface morphology of 10CrHAp and 10CrHAp-Dx coatings was obtained by atomic force microscopy (AFM) using an NT-MDT NTEGRA Probe Nano Laboratory (NT-MDT, Moscow, Russia). The scans were acquired in semi-contact mode at room temperature over 5 × 5 µm^2^ areas. The root-mean-square roughness (R_RMS_) was calculated to quantify the surface texture. Image analysis and data processing were performed with Gwyddion software (v2.59, Czech Metrology Institute, Brno, Czech Republic) [38].

#### 2.3.1. In Vitro Biological Evaluation

Data regarding the biocompatibility of the 10CrHAp and 10CrHAp-Dx coatings was obtained with the aid of human osteosarcoma MG63 cells (ATCC CRL-1427) [39], following the protocol described by Iconaru et al. [40]. The MG63 cells were cultured in Dulbecco’s Modified Eagle Medium (DMEM) previously supplemented with fetal bovine serum (FBS) at a temperature of 37 °C, and in a humidified atmosphere enriched with 5% CO_2_. The cells were seeded at a density of 1 × 10^5^ cells/well and exposed to the coatings for 24 h and 48 h. The cell viability was determined with the aid of the MTT reduction assay. For this purpose, after exposure, MTT reagent was added, and the formazan crystals that formed were solubilized. The absorbance of the medium was measured at 595 nm using a TECAN spectrophotometer (TECAN, Männedorf, Switzerland) and the percentage of viable cells was quantified relative to the untreated cell culture used as controls, which were considered 100% viable. After 4–8 h, the coatings were rinsed with sterile saline, fixed with cold methanol, and prepared for microscopic analysis to evaluate cell adhesion and proliferation on the surfaces. For the qualitative evaluation, scanning electron microscopy (SEM) and metallographic microscopy (MM) were used for the visual assessment of the adhered cells on the surfaces of the 10CrHAp and 10CrHAp-Dx coatings. Digital images were analyzed using ImageJ software (version 1.51j8) to quantify changes in cell morphology and surface coverage [37].

#### 2.3.2. Lactate Dehydrogenase (LDH) Release Measurement

Cytotoxicity was also assessed by measuring lactate dehydrogenase (LDH) release, a marker of cell membrane damage. After incubating MG63 cells with the 10CrHAp and 10CrHAp-Dx coatings for 24 h and 48 h, 50 µL of culture supernatant was collected and mixed with 50 µL of reaction mixture in a 96-well plate using a Cytotoxicity Detection KitPLUS (Roche) in agreement with the manufacturer’s instructions. After incubation at room temperature in the dark, the absorbance was measured at 485 nm using a Tecan GENios microplate reader. The relative LDH release was calculated against untreated controls, and the results were expressed as mean ± standard deviation from independent experiments.

#### 2.3.3. In Vitro Antibacterial Activity Assay

Information regarding the antibacterial properties of the 10CrHAp and 10CrHAp-Dx coatings was obtained through in vitro assays against *Pseudomonas aeruginosa* ATCC 27853 and *Staphylococcus aureus* ATCC 25923 bacterial strains. The tests were performed according to a modified protocol of Ciobanu et al. [41]. For this purpose, bacterial suspensions (5 × 10^6^ CFU/mL) were prepared in sterile conditions. Aliquots of 100 μL were applied to each coated surface and incubated at 37 °C for 24, 48, and 72 h. After each interval, the bacterial viability was determined and expressed as log CFU/mL. A positive control consisting of a free bacterial suspension (C^+^) was included. All experiments were carried out in triplicate, and the results were reported as the mean ± SD. The data were statistically analyzed using Microsoft Excel Office 365. The bacteria’s cells adhesion and colonization on the coating’s surfaces were also qualitatively examined by atomic force microscopy (AFM). After incubation, the samples were rinsed using sterile saline, fixed with cold methanol, and air-dried. The AFM imaging was conducted in non-contact mode in ambient conditions, and the topographies scans were recorder over 10 × 10 μm^2^ areas.

## 3. Results and Discussions

**Figure 1:**
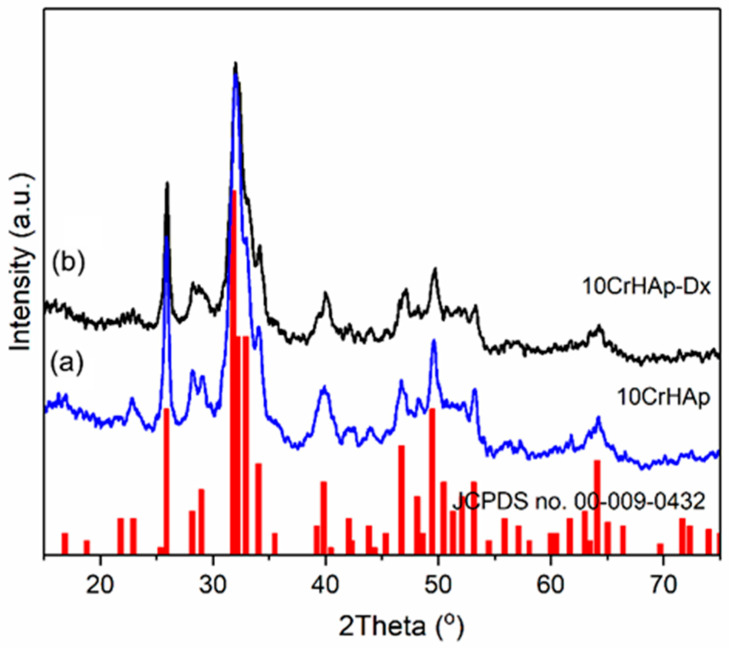
X-ray diffraction spectra of the 10CrHAp (a) and 10CrHAp-Dx (b) sample. Characteristic diffraction pattern of pure hexagonal hydroxyapatite, JCPDS No. 00-009-0432, (red bars).

In this research, X-ray diffraction (XRD) analysis was employed to determine the mineral phases and assess changes in crystallinity within the 10CrHAp and 10CrHAp-Dx coatings. Figure 1 presents the XRD spectra for the 10CrHAp (Figure 1a) and the 10CrHAp-Dx (Figure 1b) samples, alongside the reference diffraction pattern of pure hexagonal hydroxyapatite (JCPDS no. 00-009-0432). The 10CrHAp-Dx sample (Figure 1b) exhibits a slight broadening of diffraction peaks, which is indicative of reduced crystallite size and diminished crystallinity. This behavior may be attributed to the effects induced by the incorporation of dextran. The X-ray diffraction (XRD) spectra reveal prominent characteristic peaks corresponding to hexagonal hydroxyapatite (HAp) crystals, specifically associated with the (002), (102), (210), (211), (300), (202), (310), (222), (213), and (004) reflections. The lattice parameters, unit cell volume, and average crystallite size for the two analyzed samples are summarized in Table 1. The variation in crystallite size between chromium-doped hydroxyapatite (10CrHAp) synthesized with and without dextran arises from dextran’s role as a surface-active polysaccharide. By adsorbing onto the surface of growing crystallites, dextran blocks active growth sites, thereby restricting crystal growth and resulting in smaller crystallite sizes. Additionally, the surrounding dextran molecules introduce steric hindrance, which inhibits aggregation and further growth. Dextran also contributes to nanoparticle stabilization in suspension, minimizing the tendency of crystallites to agglomerate [42].

**Figure 2:**
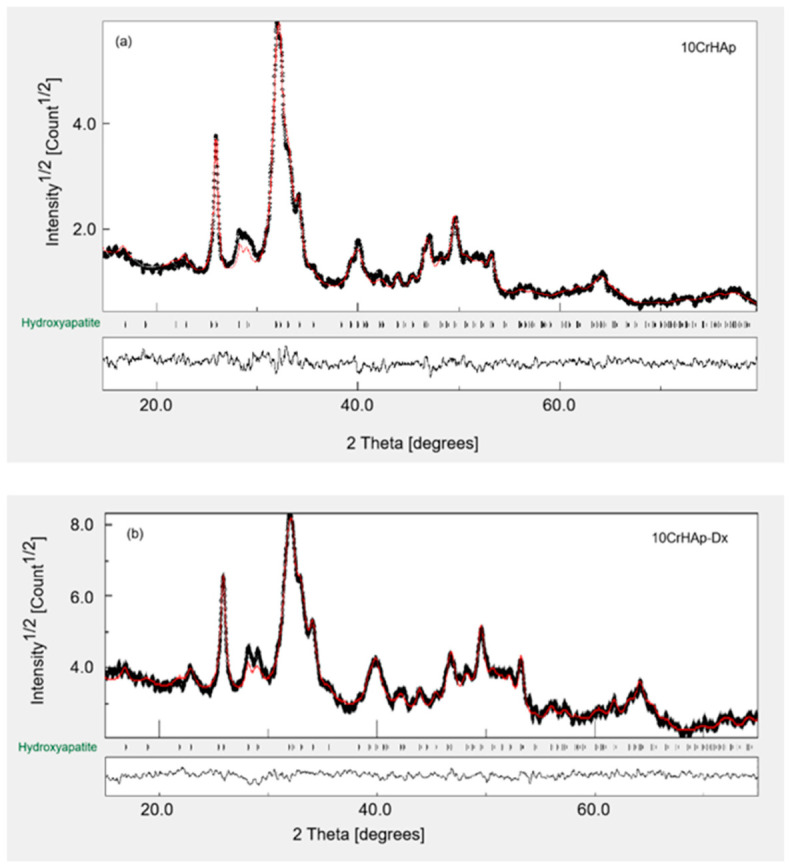
Observed (black solid lines), calculated (red solid lines) and difference (black solid lines) XRD patterns of 10CrHAp (**a**) and 10CrHAp-Dx (**b**) samples.

The observed and calculated XRD profiles of the 10CrHAp and 10CrHAp-Dx samples are shown in Figure 2, together with the corresponding difference curves. The close overlap between the experimental and calculated patterns demonstrates the successful convergence of the Rietveld refinement, as evidenced by the minimal residuals. Both samples exhibit the characteristic reflections of hexagonal hydroxyapatite (HAp), indexed to the P6_3_/m space group and consistent with JCPDS card No. 00-009-0432. No secondary crystalline phases or dextran-related diffraction features were detected, indicating that chromium incorporation and subsequent dextran loading do not disrupt the apatite structure. This phase purity is further supported by the refinement indicators (Rwp ≈ 6.3%, Rp ≈ 4.9–5.0%, χ^2^ ≈ 0.22), which confirm the high quality of the fits. The goodness-of-fit values, Rwp factors, refined lattice parameters, crystallite sizes, and R-values for both samples are summarized in Table 2.

The refined structural parameters reveal a systematic increase in both lattice constants (a and c) relative to reference HAp (a = 9.418 Å, c = 6.884 Å, and V = 528.08 Å^3^). For 10CrHAp, the lattice expands slightly to a = 9.423 Å and c = 6.889 Å, yielding a unit cell volume of 529.730 Å^3^. This modest expansion is consistent with the incorporation of Cr^3+^ ions into Ca^2+^ sites. Although Cr^3+^ possesses a smaller ionic radius than Ca^2+^, the substitution typically induces local charge compensation (e.g., OH^−^ vacancies, protonation, or lattice relaxation), which can lead to a net increase in cell volume. Dextran loading produces a more pronounced lattice expansion. The 10CrHAp-Dx sample exhibits a = 9.429 Å and c = 6.907 Å, with the unit cell volume increasing to 531.804 Å^3^. The preferential elongation along the c axis (Δc ≈ +0.018 Å relative to 10CrHAp) suggests that dextran interacts with the hydroxyl channels or surface phosphate groups, generating local distortions. Such effects are consistent with hydrogen bonding, partial intercalation, or strong surface adsorption of dextran, all of which can relax the apatite lattice. A reduction in crystallite size is also observed upon dextran loading, decreasing from 23.36 nm for 10CrHAp to 19.83 nm for 10CrHAp-Dx. This decreases, accompanied by slight peak broadening and a marginal increase in Rp, indicates that dextran inhibits crystal growth or enhances surface disorder. These microstructural changes are characteristic of polymer-modified HAp systems and support the presence of strong organic–inorganic interactions between dextran and the Cr-doped HAp surface.

It was found that the heat treatment at 80 °C for 72 h is sufficient to obtain crystalline HAp films, since the hydroxyapatite phase was already formed during the synthesis step performed at the same temperature. The nucleation and initial growth of the crystals take place in suspension, so that the spin-coated layers contain pre-crystallized HAp nanoparticles. Under these conditions, the film obtained by spin coating does not contain an amorphous precursor that would require a higher temperature for crystallization, but pre-formed HAp particles with a stable crystalline structure. Therefore, the post-deposition treatment mainly serves to remove residual water and organic substances, to promote particle consolidation and to allow the limited maturation of the crystals. The prolonged exposure at 80 °C provides an adequate thermal activation for these processes, allowing the preservation and a slight increase in crystallinity without requiring an annealing at a higher temperature that could damage the polymer or composite substrate.

**Figure 3:**
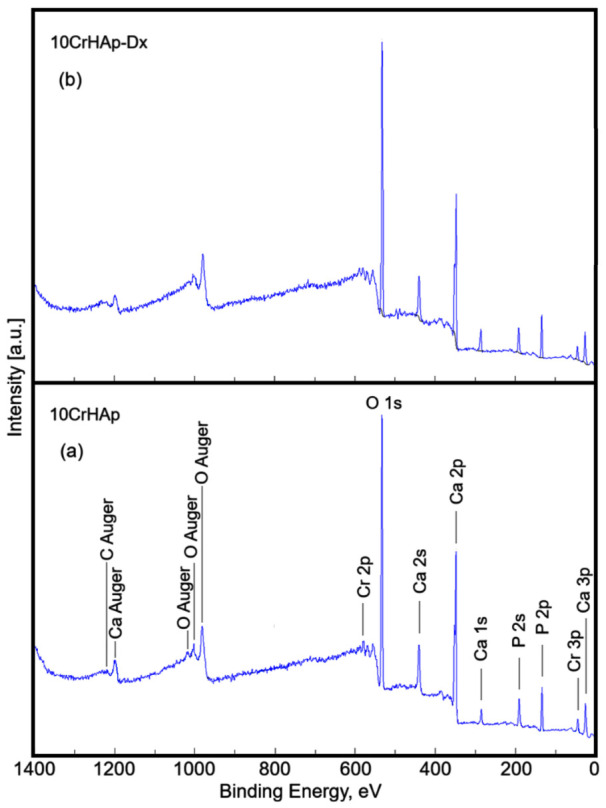
Survey scan XPS spectra of the 10CrHAp (**a**) and 10CrHAp-Dx (**b**) thin films recorded at room temperature.

In this study, the surface composition of 10CrHAp and 10CrHAp-Dx films was determined by XPS. The survey scan XPS spectra of the 10CrHAp and 10CrHAp-Dx samples are presented in Figure 3. The observed peaks correspond to the elemental components of each material such as oxygen, calcium, phosphorus, chromium. The carbon observed in the 10CrHAp sample is due to contamination, while in the 10CrHAp-Dx sample we can observe carbon due to the presence of the dextran matrix.

**Figure 4:**
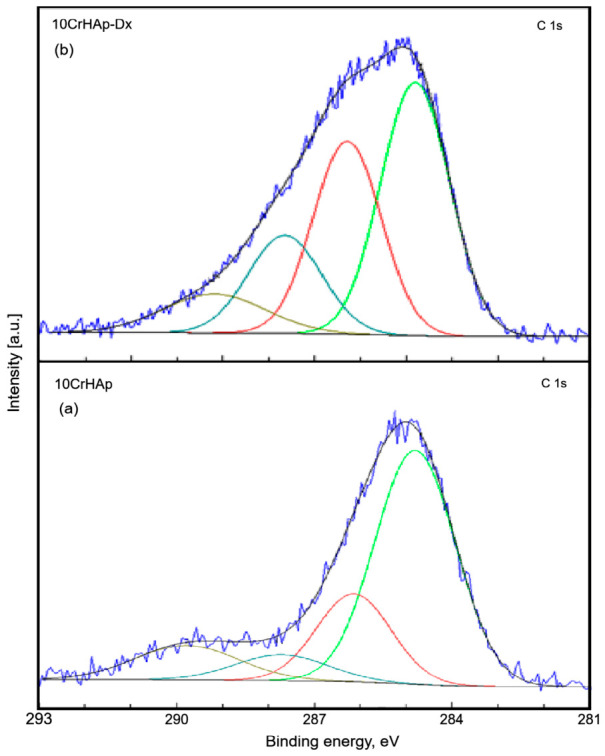
XPS high-energy resolution spectra of C 1s peaks for 10CrHAp (**a**) and 10CrHAp-Dx (**b**) samples.

A least-square fitting procedure with a Gaussian–Lorentzian product function was used to decompose the peaks observed in the XPS spectra of the two samples. The high-resolution spectra of the constituent elements of the two samples revealed the detailed chemical states. The C 1s band of both samples was decomposed into four components (Figure 4a,b). The first component was noticed at 284.8 eV for both samples. The second component was detected at 286.14 (10CrHAp) and 286.29 eV (10CrHAp-Dx). The third component was identified at 287.72 (10CrHAp) and 287.65 eV (10CrHAp-Dx), while the fourth component was localized at 289.78 (10CrHAp) and 289.19 eV (10CrHAp-Dx). The first component is typical of carbon bonded only to carbon and hydrogen [C–(C, H)]. The second component can be attributed to C–O single bonds. The third component is typical of C=C and O–C–O bonds [43,44,45]. The fourth component was attributed to contaminants containing –COOR groups.

**Figure 5:**
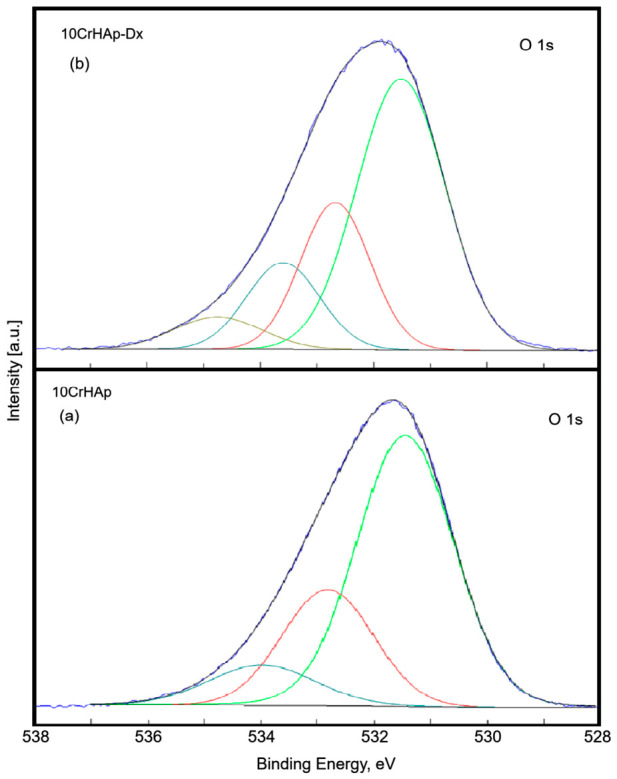
XPS high-energy resolution spectra of O 1s peaks for 10CrHAp (**a**) and 10CrHAp-Dx (**b**) samples.

The O 1s peak for both samples (Figure 5a,b) was deconvoluted into three components for 10CrHAp and four components for 10CrHAp-Dx. The first component appeared at 531.49 eV (10CrHAp) and 531.51 eV (10CrHAp-Dx), corresponding to oxygen atoms in hydroxyapatite, the PO_4_^3−^ groups of HAp, and C=O double bonds. The second component was detected at 532.82 eV (10CrHAp) and 532.68 eV (10CrHAp-Dx), and was attributed to C–O, C–OH, and O–C–O chemical bonds associated with the presence of polymer [45]. The third component, observed at 533.98 eV (10CrHAp) and 533.60 eV (10CrHAp-Dx), was linked to residual adsorbed water and potential contaminants containing C–O single bonds. Additionally, the 10CrHAp-Dx sample exhibited a fourth component at 534.76 eV, which significantly deviates from the typical literature values. This behavior may indicate the presence of a region with altered charge distribution, likely influenced by the incorporation of dextran.

**Figure 6:**
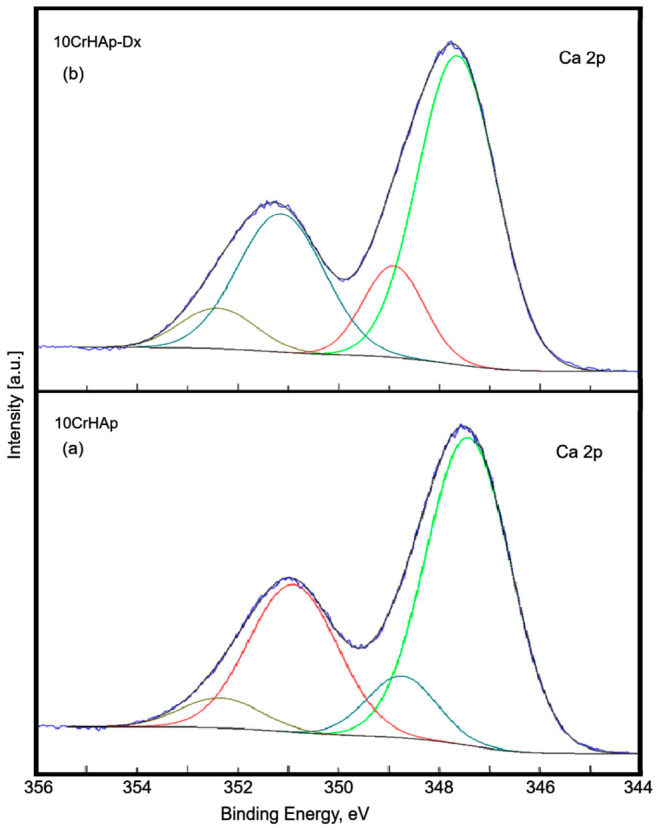
XPS high-energy resolution spectra of Ca 2p peaks for 10CrHAp (**a**) and 10CrHAp-Dx (**b**) samples.

Figure 6 displays the high-resolution Ca 2p XPS spectra for the 10CrHAp and 10CrHAp-Dx samples. Spectral deconvolution revealed four distinct components in both samples. In the 10CrHAp sample, a primary doublet was identified at binding energies (BE) of 347.36 eV (2p_3_/_2_) and 350.96 eV (2p_1_/_2_), with a characteristic energy separation of approximately 3.6 eV and an area ratio close to 2:1. The primary doublet in the 10CrHAp-Dx sample appeared at slightly higher BEs of 347.58 eV (2p_3_/_2_) and 351.18 eV (2p_1_/_2_). Additionally, both samples exhibited a secondary doublet: 348.77 eV and 352.35 eV for 10CrHAp, and 348.82 eV and 352.42 eV for 10CrHAp-Dx, maintaining the same 3.6 eV separation and 2:1 intensity ratio.

**Figure 7:**
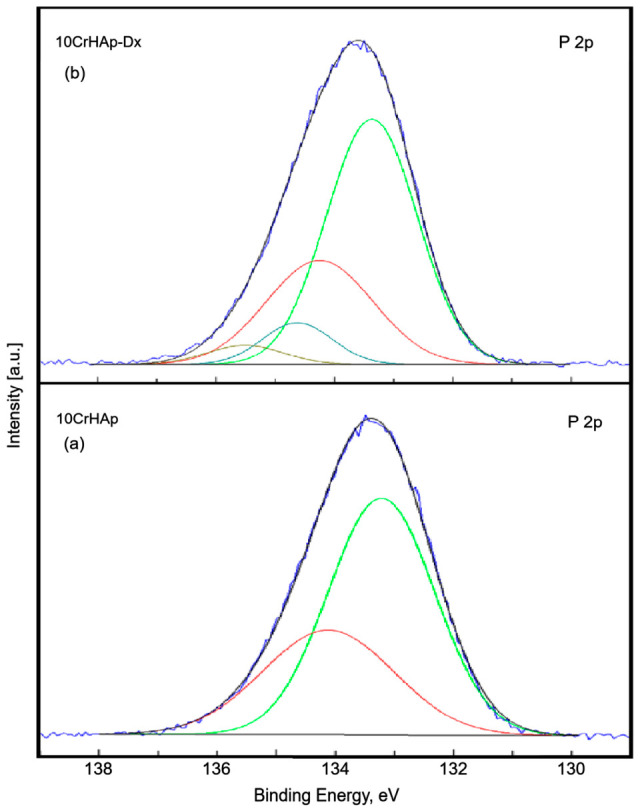
XPS high-energy resolution spectra of P 2p peaks for 10CrHAp (**a**) and 10CrHAp-Dx (**b**) samples.

In Figure 7a the high-resolution XPS spectrum of P 2p of the 10CrHAp sample is presented. The P 2p doublet with the two specific lines (2p3/2 and 2p1/2) at binding energies of 133.24 and 134.14 eV is observed. The two lines are spaced at approximately 0.9 eV and have an area ratio close to 2:1. The binding energy of the doublet is specific to hydroxyapatite (–PO4), in accordance with previous studies [46]. Figure 7b shows the high-resolution XPS spectrum of P 2p of the 10CrHAp-Dx sample. The high-resolution XPS spectrum of the 10CrHAp-Dx sample shows four distinct components. The first doublet appears at 133.37 eV and 134.27 eV and corresponds to P2p3/2 and P2p1/2. The two lines are separated by about 0.9 eV and the area ratio is about 2:1. The binding energy of this doublet is specific to phosphate in HAp [46]. The second doublet was located at 134.63 eV and 135.51 eV. This doublet has a higher energy suggesting the presence of phosphate in a more oxidized state.

**Figure 8:**
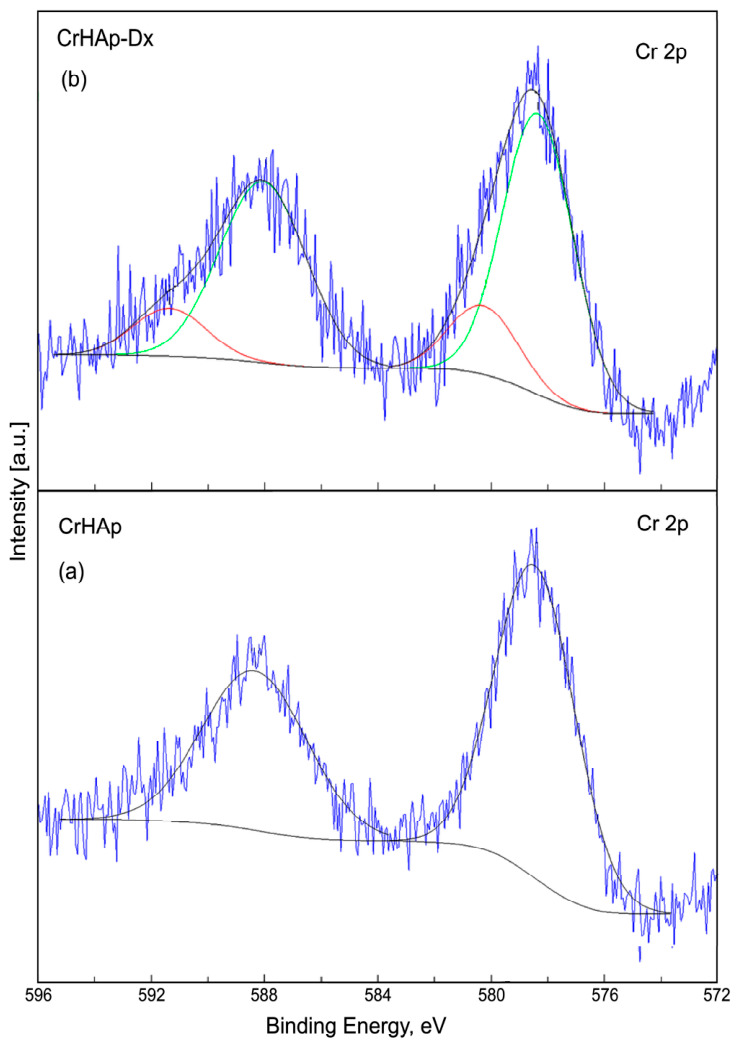
XPS high-energy resolution spectra of Cr 2p peaks for 10CrHAp (**a**) and 10CrHAp-Dx (**b**) samples.

The high-resolution XPS spectrum of Cr 2p of 10CrHAp shown in Figure 8a showed two distinct components. The first component was observed at BE of 578.15 and the second component at BE of 588.13 eV. The first component corresponds to the Cr 2p_3/2_ orbital. The second component is associated with the Cr 2p_1/2_ orbital. The high-resolution XPS spectrum of Cr 2p of 10CrHAp-Dx (Figure 8b) revealed four distinct components. The first component was observed at BE of 578.10 (Cr 2p_3/2_) and the second component at BE of 588.09 eV (Cr 2p_1/2_). The separation between the Cr 2p_3/2_ and Cr 2p_1/2_ peaks for both analyzed samples was 9.98 eV (10CrHAp) and 9.99 eV (10CrHAp-Dx), respectively. The results are in agreement with previous XPS studies [47,48], which showed that the separation between the Cr 2p_3/2_ and Cr 2p_1/2_ peaks is usually around 9–10 eV. The other two components of the high-resolution XPS spectrum of Cr 2p of 10CrHAp-Dx were located at BE of 588.15 and 591.46 eV. In the case of the two analyzed samples, the peaks corresponding to the Cr 2p_3/2_ orbital are often associated with the presence of chromium in the +3 oxidation state.

Moreover, Table 3 reveals the chemical composition of 10CrHAp and 10CrHAp-Dx samples obtained from XPS studies.

**Figure 9:**
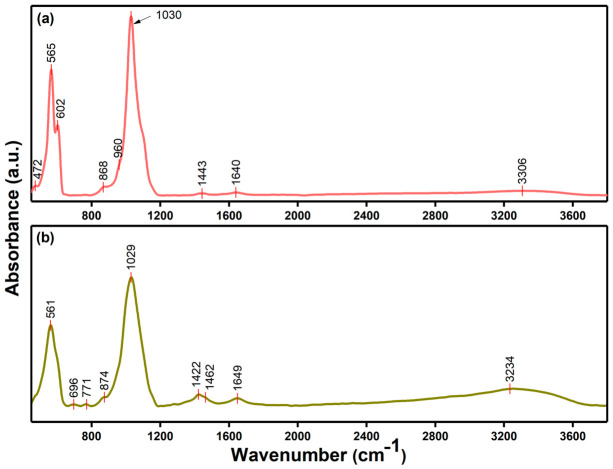
FTIR general spectra obtained for 10CrHAp (**a**) and 10CrHAp-Dx (**b**) coatings in the 450–3800 cm^−1^ spectral domain.

Fourier-transform infrared (FTIR) spectroscopy was employed to identify the functional groups and to assess the chemical structure of 10CrHAp and 10CrHAp-Dx. The corresponding FTIR spectra are shown in Figure 9.

The FTIR spectra obtained for the 10CrHAp (Figure 9a) in the 450–3800 cm^−1^ spectral domain reveals the presence of two intense vibrational bands centered at 565 cm^−1^ and 1030 cm^−1^, corresponding to the ν_4_ and ν_3_ vibrational modes of the phosphate (PO_4_^3−^) groups within the apatite structure [49]. In the same FTIR spectra are observed maxima that are centered at 472 cm^−1^ (ν_2_ bending mode of PO_4_^3−^), 602 cm^−1^ (ν_4_ bending mode of PO_4_^3−^), and 960 cm^−1^ (ν_1_ symmetric stretching of PO_4_^3−^), which belong to the apatite structure [49]. At around 1443 cm^−1^, a broad maximum that could be attributed to the vibration of carbonate groups [49] could be observed. The vibration band attributed to carbonate groups likely arises from surface-adsorbed impurities rather than structural incorporation. Such species may originate from synthesis, washing, or handling steps, as hydroxyapatite surfaces readily capture atmospheric or solution-borne carbonates. This behavior is widely reported for HAp-based materials due to the strong affinity of Ca^2+^ for CO_3_^2−^ in aqueous media [50,51]. In the CrHAp-Dx composite, the polymer matrix can further promote ion exchange and the adsorption of dissolved carbonate species, enhancing the intensity of these bands. Importantly, this type of superficial contamination is common and does not alter the crystalline structure of the material. The FTIR observations are fully consistent with the XPS results, which also indicate the presence of surface carbonaceous species rather than lattice substitution. Moreover, vibrational maxima observed at approximately 1640 cm^−1^ and 3306 cm^−1^, corresponding to the H–O–H bending vibration and the O–H stretching vibration, respectively, are also present in the FTIR spectra of 10CrHAp [49].

In the case of the FTIR spectra of 10CrHAp-Dx (Figure 9b), it is noticed that the addition of dextran induces a broadening of the absorption maxima accompanied by a decrease in their intensity. Additionally, it is observed that dextran causes a shift in the characteristic 10CrHAp bands toward lower wavenumbers suggesting possible molecular interactions between the components. Similar FTIR spectral modifications—band broadening, intensity reduction, and shifts toward lower wavenumbers—have been reported in dextran-coated hydroxyapatite systems, confirming molecular interactions between the organic and inorganic phases [52].

On the other hand, in the FTIR spectrum of 10CrHAp-Dx, in addition to the vibrational bands characteristic of the 10CrHAp structure, absorption maxima that indicate the presence of dextran are also observed (Figure 9b). The vibrational maxima associated with the dextran structure are predominantly observed within the following spectral regions: 1200–1500 cm^−1^ (corresponding to symmetrical CH_2_ deformations and C–OH bending modes), 950–1200 cm^−1^ (attributed to C–O stretching vibrations) and 750–950 cm^−1^ (α-glucopyranose ring deformation) [49,53,54,55]. Accordingly, the absorption bands identified at approximately 771 (α-glucopyranose ring deformation), 1422 cm^−1^ (ν(C–H) and δ(C–H)) in the FTIR spectra are characteristic of polysaccharide (dextran) components [49,53,54,55]. The bands that appear in the 1400–1500 cm^−1^ region are commonly associated with C–H deformation or C–O–H vibrational modes originating from saccharide structures (such as dextran) [49,53,54,55]. Additionally, the broad peak detected in the 3100–3500 cm^−1^ spectral region could be attributed to the stretching vibration of adsorbed water molecules (O–H stretching vibration) [49,53,54,55].

Similar FTIR findings have been reported in earlier studies conducted by Predoi, D. et al. [49], where the incorporation of dextran into hydroxyapatite-based systems led to noticeable spectral modifications. Specifically, a broadening of the phosphate vibrational bands (ν_1_, ν_3_, and ν_4_ modes) was reported, accompanied by a decrease in their intensity and a shift toward lower wavenumbers of the maxima position. These changes were attributed to molecular interactions between the dextran chains and the hydroxyapatite matrix, likely involving hydrogen bonding and surface adsorption phenomena [49]. Such spectral behavior reinforces the interpretation that dextran integration alters the local chemical environment of the inorganic phase, consistent with the observations in the present study.

**Figure 10:**
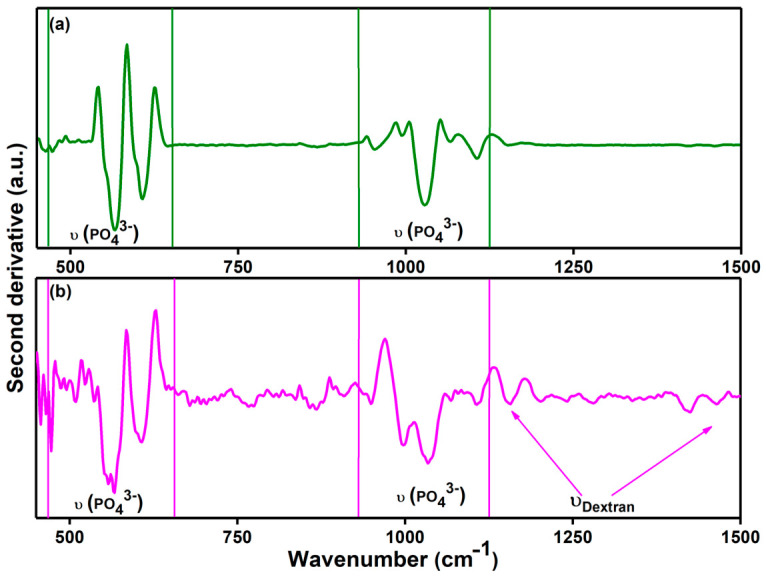
FTIR second derivative spectra obtained for 10CrHAp (**a**) and 10CrHAp-Dx (**b**) coatings in the 450–1500 cm^−1^.

The FTIR second derivative spectra obtained for the 10CrHAp and 10CrHAp-Dx coatings in the 450–1500 cm^−1^ region are depicted in Figure 10. Usually, maxima appear in this spectral domain that are characteristic to apatite and dextran structure.

In the second derivative FTIR spectra recorded between 450 and 1500 cm^−1^, distinct vibrational characteristic corresponding to phosphate and saccharide groups are clearly observed. For chrome-doped hydroxyapatite (10CrHAp), sharp negative peaks corresponding to the ν_4_ bending, ν_1_ symmetric stretching, and ν_3_ asymmetric stretching modes of PO_4_^3−^ are observed. These well-defined signals reflect the crystallinity and structural integrity of the 10CrHAp phase. Upon the incorporation of dextran (10CrHAp-Dx), the obtained FTIR second derivative spectra reveal some spectral changes. The phosphate bands become broader, with a reduced intensity and slight shifts toward lower wavenumbers, indicating a decrease in crystallinity and possible molecular interactions between dextran and the 10CrHAp surface. Additionally, new absorption peaks which are attributed to C–H bending and C–O stretching vibrations from the dextran backbone appear. These changes confirm the presence of dextran in the 10CrHAp-Dx and could suggest the formation of hydrogen bonds or electrostatic interactions between the organic and inorganic components.

**Figure 11:**
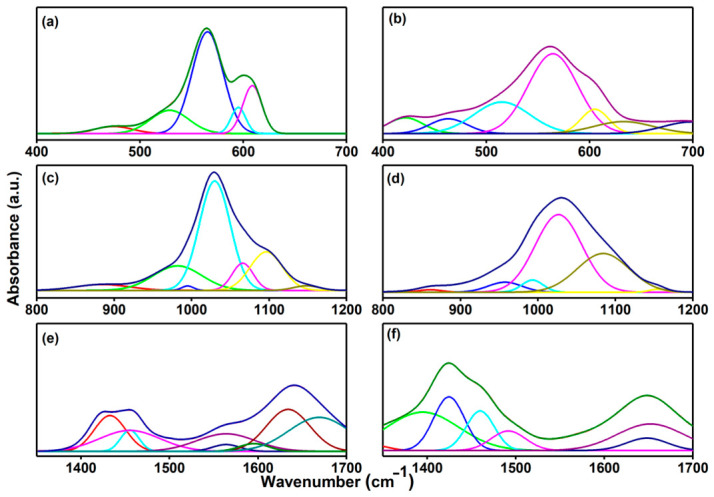
FTIR deconvoluted spectra obtained for 10CrHAp (**a**,**c**,**e**) and 10CrHAp-Dx (**b**,**d**,**f**) coatings in the 400–700 cm^−1^, 800–1200 cm^−1^ and 1300–1700 cm^−1^.

The deconvoluted FTIR spectra of 10CrHAp and 10CrHAp-Dx coatings were analyzed in the 400–700 cm^−1^, 800–1200 cm^−1^ and 1300–1700 cm^−1^ regions to investigate the structural and chemical characteristics of the materials (Figure 11). In the 400–700 cm^−1^ range, the vibrations of the phosphate groups (PO_4_^3−^) and the hydroxyl librational modes usually appear, with well-defined bands in 10CrHAp indicating preserved crystallinity, while slight broadening and shifts in 10CrHAp-Dx suggest surface interactions and minor lattice distortions due to polymer incorporation (Figure 11a,b) [19,52]. Also, the 800–1200 cm^−1^ domain is usually dominated by the ν_1_ and ν_3_ phosphate stretching modes, revealing clear differences between the two samples: 10CrHAp showed sharp peaks characteristic of a well-ordered apatite network, while 10CrHAp-Dx showed additional bands and subtle shifts corresponding to the C–O and C–O–C vibrations of dextran, indicating the polymer adsorption and possible hydrogen bonding with the phosphate groups (Figure 11c,d) [52,56,57,58,59].

Finally, in the 1300–1700 cm^−1^ region, bands associated with CH_2_/CH_3_ deformation, carbonates and water bending were detected; the 10CrHAp-Dx sample showed increased intensity of the organic-related bands and minor shifts compared to 10CrHAp, confirming the presence of dextran and its interaction with the 10CrHAp, while the carbonate and water signals reflected network substitutions and surface hydration (Figure 11e,f) [19]. Overall, the combined spectral analysis, supported by deconvolution and second derivative analysis, confirms the coexistence of 10CrHAp and dextran, highlights their structural interactions, and provides information on the changes in crystallinity and surface chemistry induced by polymer incorporation. Moreover, the FTIR results clearly indicate that the addition of the polymer (dextran) induces band broadening and a decrease in their intensity, as well as a slight shift in their position.

Furthermore, in the 400–700 cm^−1^ spectral domain a good fit for the 10CrHAp was obtained using five sub-bands that correspond to the typical phosphate vibrations of the apatite lattice, confirming its structure. In contrast, the 10CrHAp-Dx required a larger number of sub-bands (eight), which indicates a more complex vibrational pattern. In the 800–1200 cm^−1^ region, a satisfactory fit for 10CrHAp was achieved using seven sub-bands. In contrast, six sub-bands were required to obtain a good fit for the 10CrHAp–Dx coatings in this spectral region. In the 1300–1700 cm^−1^ spectral region, eight sub-bands were used to achieve a satisfactory fit for the 10CrHAp sample. In comparison, seven sub-bands were employed for the deconvolution of the 10CrHAp–Dx FTIR spectra in the same region. This complexity arises from the interaction between chromium-doped hydroxyapatite and dextran (Dx) molecules, leading to additional vibrational contributions from the polymer functional groups and possible structural distortions or substitutions within the apatite lattice.

**Figure 12:**
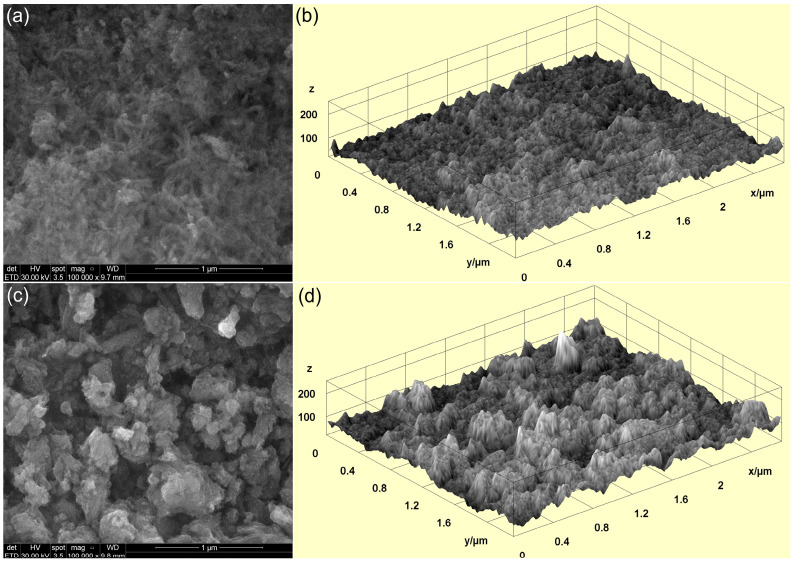
SEM images (2D and 3D representation) obtained on 10CrHAp (**a**,**b**) and 10CrHAp-Dx (**c**,**d**) coatings.

To obtain a comprehensive understanding of the surface morphology of the 10CrHAp and 10CrHAp-Dx coatings, scanning electron microscopy (SEM) analyses were conducted. Both two-dimensional (2D) and three-dimensional (3D) SEM micrographs (Figure 12) were obtained, providing high-resolution insights into the topography and surface texture of the 10CrHAp and 10CrHAp-Dx coatings. Firstly, the SEM data confirm that both coatings exhibit well-structured surfaces. Furthermore, in the obtained SEM image (both 2D and 3D representation), it is noticeable that the coatings surfaces consist of the aglomeration of nanometric particles distributed unevenly across their surface. Notably, the 10CrHAp-Dx coatings (Figure 12c,d) display a more pronounced particle agglomeration, indicating that the incorporation of dextran affects the clustering of 10CrHAp nanoparticles. This increased agglomeration may also influence the coatings’ surface roughness, potentially enhancing cell adhesion features.

**Figure 13:**
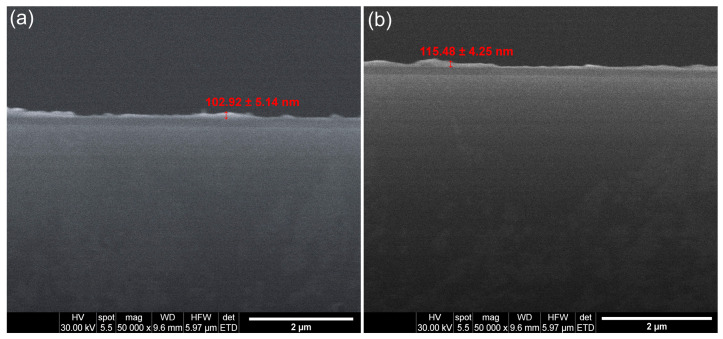
SEM transversal cross-section of 10CrHAp (**a**) and 10CrHAp-Dx (**b**) coatings.

The SEM transversal cross-section analysis provided preliminary information regarding the thickness of the coatings (10CrHAp and 10CrHAp-Dx). The results of these measurements are presented in Figure 13. The results indicate a relatively uniform layer thickness across the sample, suggesting a consistent deposition process. The SEM transversal cross-section measurements showed that the coatings’ thickness was approximately 102.92 ± 5.14 nm (for 10CrHAp; Figure 13a) and around 115.48 ± 4.25 nm for 10CrHAp-Dx (Figure 13b). Furthermore, the obtained SEM transversal cross-section images of both coatings demonstrates that the spin coating method allows coatings with a nearly uniform thickness across their surface to be obtained.

**Figure 14:**
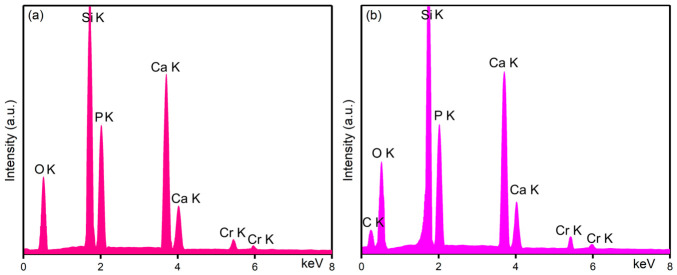
EDX spectra illustrating the elemental composition of 10CrHAp (**a**) and 10CrHAp-Dx (**b**) coatings.

Figure 14 depicts the results of the X-ray energy-dispersive spectroscopy (EDX) studies conducted on 10CrHAp and 10CrHAp-Dx coatings. EDX provides qualitative information about the elemental composition of 10CrHAp and 10CrHAp-Dx coatings.

In the case of 10CrHAp coatings (Figure 14a), only the characteristic lines corresponding to the main constituent elements calcium, phosphorus, oxygen, and chrome are observed in the spectra. In the 10CrHAp-Dx spectra, the presence of an additional line attributed to the carbon together with the Cr, Ca, P and O lines confirms the incorporation of dextran into the samples.

In the both EDX spectra (Figure 14a,b) a line attributed to the Si appears, which originates from the coating’s substrate. For 10CrHAp and 10CrHAp-Dx, their characteristic EDX spectra confirm the expected composition of the coatings. The absence of supplementary lines in both EDX spectra indicates the purity of the 10CrHAp and 10CrHAp-Dx coatings.

Additional information about the 10CrHAp and 10CrHAp-Dx samples chemical composition were obtained from EDX semi-quantitative analysis. The obtained data provide valuable insight into the chemical composition of the sample, and are in good agreement with the XPS findings. The results of the EDX semi-quantitative analysis are summarized and presented in Table 4.

The results of the adhesion test performed on the 10CrHAp and 10CrHAp-Dx coatings suggest that 10CrHAp-Dx coatings exhibit improved adhesion compared to 10CrHAp coatings. Overall, both samples exhibit a good adherence to the Si substrate (the removed adhesive tape was almost clean, with only a negligible amount of material transferred to it). These findings suggest that the spin coating method is an efficient technique for obtaining 10CrHAp-based coatings with good adhesion to Si substrates.

**Figure 15:**
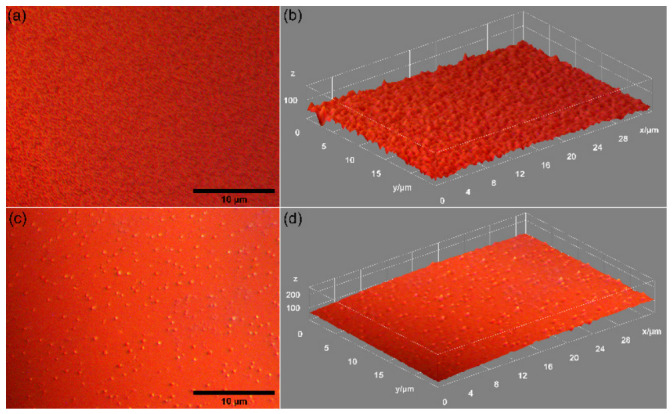
MM images (2D and 3D representation) obtained on 10CrHAp (**a**,**b**) and 10CrHAp-Dx (**c**,**d**) coatings.

The MM images of 10CrHAp and 10CrHAp-Dx coatings surfaces obtained at 50× magnification is revealed in Figure 15. Our MM results underline that both 10CrHAp and 10CrHAp-Dx coatings exhibit well-structured, continuous, and homogeneous surfaces. On the other hand, the MM images reveal that all the analyzed surfaces are free of cracks, fissures, or other defects, indicating that the spin coating method allows for the deposition of coatings with good surface characteristics. Moreover, the MM results suggest that the incorporation of dextran (Dx) in 10CrHAp-Dx does not compromise the uniformity or continuity of the developed coating.

**Figure 16:**
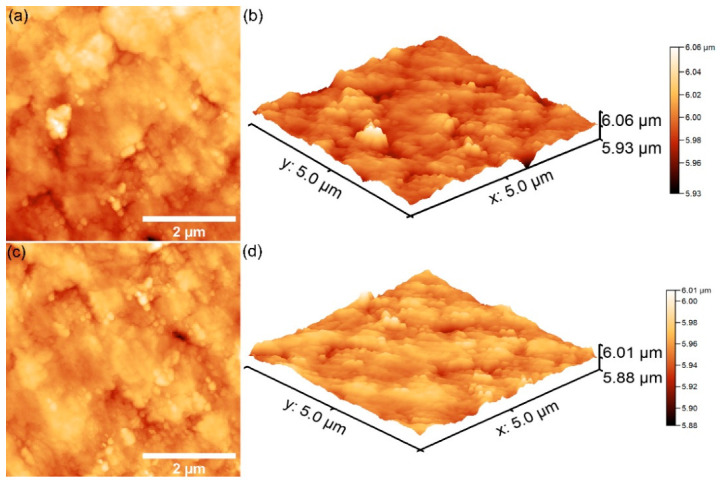
Two-dimensional AFM topographies of 10CrHAp (**a**) and 10CrHAp-Dx (**b**) coatings’ surfaces acquired on an area of 5 × 5 µm^2^ and their corresponding 3D representations (**c**,**d**).

The surface features of the 10CrHAp and 10CrHAp-Dx coatings were studied using atomic force microscopy (AFM). The surface scans were performed at room temperature and normal atmospheric conditions in semi-contact mode over an area of 5 × 5 μm^2^, and the corresponding AFM images are shown in Figure 16.

The AFM topographies in Figure 16a, depicting the surface of 10CrHAp coatings, reveal a continuous and uniform layer with a generally smooth and homogeneous appearance. Both the 2D as well as the 3D representations (Figure 16b) showed evenly distributed clusters of small (nanometer-sized) particles across the surface. Furthermore, the AFM results showed that no major defects, cracks, or irregularities were visible, which indicates that the deposition process produced a stable and consistent coating. This kind of smooth and defect-free surface is often desirable because it suggests good coating quality and reliable performance. On the other hand, the AFM topography of the surface of the 10CrHAp-Dx coating, displayed some noticeable differences (Figure 16c). Due to the presence of dextran matrix, the coating’s surface appeared even more uniform and compact. In the 2D AFM images (Figure 16c), particle clusters could be clearly distinguished, and these clusters appear to be larger and more evenly spread than those noticed on the surface of 10CrHAp coating. The 3D representation of AFM topography of 10CrHAp-Dx (Figure 16d) highlighted the continuous nature of the coating but with more pronounced surface features. These changes can be attributed to the role of the dextran polymer matrix, which seems to encourage the particles to arrange themselves in a more ordered way. As a result, the surface becomes not only homogeneous but also slightly rougher, which may influence how the coating interacts with different environments. To better understand these surface changes, the roughness of each coating was quantified using the R_RMS_ parameter. For the 10CrHAp coating an R_RMS_ value of 16.64 nm, was obtained, while for the 10CrHAp-Dx coating a higher value of 23.18 nm was determined. This increase in roughness suggests that the presence of a dextran matrix could lead to a surface with more pronounced features. From a practical point of view, such an increase in roughness could be beneficial, since slightly rougher surfaces often provide better conditions for cell attachment and growth, which is particularly important in biomedical applications.

The AFM results demonstrate that while both coatings exhibited a good uniformity and stability, the presence of the dextran matrix from the 10CrHAp-Dx coatings helped to improve surface homogeneity and increase roughness in a controlled way. The 10CrHAp coating displayed a smooth, homogeneous structure with evenly distributed nanometric clusters, whereas the presence of a dextran matrix in 10CrHAp-Dx further improved surface uniformity and promoted the development of more pronounced and regularly distributed agglomerates. These combined effects could lead to an enhancement of the biological performance of the coatings, making 10CrHAp-Dx especially promising for being used in biomedical implants and tissue regeneration.

The results obtained from various microscopy techniques (such as MM, SEM, and AFM analyses) clearly demonstrate that the spin coating method used to fabricate the 10CrHAp and 10CrHAp-Dx coatings reliably produces surfaces that are uniform, and continuous. This approach ensures that the coatings are free from significant defects such as cracks, fissures, or irregularities, highlighting the precision and reproducibility of the deposition process. Moreover, the consistency observed across different imaging methods confirms that the method is highly effective for creating high-quality, defect-free coatings suitable for applications where surface integrity and homogeneity are critical.

**Figure 17:**
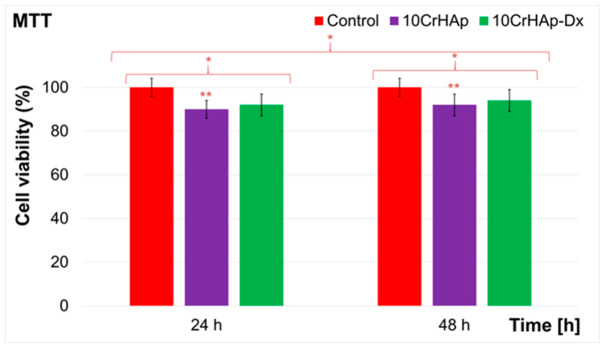
Cell viability of MG63 cells incubated with 10CrHAp and 10CrHAp-Dx for 24 and 48 h. The results are represented as the mean ± standard deviation (SD) and are expressed as percentages of control (100% viability). Statistical analysis was performed using one-way ANOVA followed by a post hoc Student’s *t*-test. Significance levels are indicated as follows: * *p* = 0.01; ** *p* = 0.04; and *p* ≥ 0.05 were considered non-significant.

The cytocompatibility of the 10CrHAp and 10CrHAp-Dx coatings was evaluated using the MTT colorimetric assay using the MG63 cell line. The cell viability was measured after 24 h and 48 h of incubation and expressed as the mean ± SD relative to the untreated control (100% viability). The results are presented in Figure 17.

The results of the MTT assay revealed that both investigated coatings systems exhibited high levels of cell viability, confirming their good biocompatibility in accordance with ISO 10993-1:2018 guidelines [60], which consider that cell viability values above 88% are indicative of non-cytotoxic materials. For the 10CrHAp coatings, the viability values remained consistently above 88%, with ~90% after 24 h and ~92% after 48 h of incubation, thus indicating that the coating’s surface provided a stable environment for the MG63 cells’ attachment and proliferation. Furthermore, the MTT assay findings highlighted that the presence of the dextran matrix further improved the biological response of the coatings. The 10CrHAp-Dx coatings exhibited cell viability values of approximately 92% at 24 h, which increased to ~94% after 48 h, therefore surpassing the corresponding values obtained in the case of 10CrHAp coatings. Although the differences in cell viability appear to be slight, they were reproducible and highlight the beneficial contribution of dextran to the composite coating. The slightly higher viability associated with 10CrHAp-Dx coatings can be attributed to several factors linked to the presence of dextran. Dextran is a highly hydrophilic polysaccharide that enhances surface wettability and supports the formation of a hydrated layer at the material interface, facilitating nutrient and oxygen diffusion and promoting a favorable environment for cell adhesion and metabolic activity [61,62]. More than that, it has been reported that polysaccharide matrices are able to modulate protein adsorption in order to maintain bioactive conformations of adhesion molecules such as fibronectin and vitronectin, thereby supporting integrin-mediated cell attachment and proliferation [63]. Another possible mechanism is that the dextran matrix reduces could help reduce the local ionic stress. While hydroxyapatite dissolution products are generally bioactive, the excessive ionic fluctuations may pose a threat to the cells; therefore, embedding the ceramic within a dextran network may act as a buffer to such effects and provide a more stable microenvironment [64]. Additionally, the inclusion of polymeric components can soften the otherwise rigid ceramic interface, creating a more biomimetic mechanical environment that enhances osteoblast spreading and viability [65]. Taking these aspects into consideration, the MTT assay results demonstrate that both 10CrHAp and 10CrHAp-Dx coatings possess excellent cytocompatibility with MG63 cells, with cell viability consistently maintained above 90% after 48 h. More than that, the data suggested that the 10CrHAp-Dx exhibited a noticeable improvement in cell viability, suggesting that the polysaccharide matrix contributes to a more supportive and bioactive surface. These results emphasize the potential of 10CrHAp-Dx coatings as promising candidates for biomedical applications requiring enhanced osteoblast viability and long-term performance.

**Figure 18:**
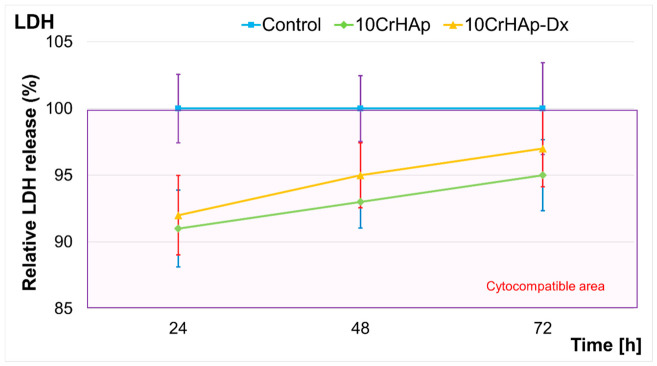
Lactate dehydrogenase (LDH) release (%) from cells cultured with control and 10CrHAp and 10CrHAp-Dx coatings over 24, 48, and 72 h. LDH release was expressed relative to untreated control cells (set at 100%). Data are presented as the mean ± standard deviation. The shaded region (85–100%) represents the cytocompatible range.

The cytocompatibility of the 10CrHAp and 10CrHAp-Dx coatings were also evaluated using the lactate dehydrogenase (LDH) release assay over 24, 48, and 72 h of incubation. The control group maintained a constant relative LDH release of 100% across all time points, serving as the baseline reference. The results of the LDH assay are depicted in Figure 18. The LDH assay revealed that both types of coatings remained well within the cytocompatible area (highlighted in the purple-shaded region), indicating that neither the 10CrHAp nor the 10CrHAp-Dx induced significant cytotoxic effects to the MG63 cells.

The LDH assay studies membrane integrity by quantifying the release of the cytoplasmic enzyme LDH into the culture medium. The fact that both 10CrHAp and 10CrHAp-Dx consistently showed LDH release values that were below the 100% control baseline confirms that coatings do not compromise cell membrane integrity and could be considered cytocompatible. The slight increase observed in the relative LDH release over time for both coatings groups is expected and likely reflects a natural, gradual accumulation of LDH from normal cell turnover during the prolonged time of culture, rather than a material-induced toxicity. The increase in LDH release observed for 10CrHAp-Dx compared to 10CrHAp may be attributed to the presence of dextran which could exert a mild influence on cellular metabolism or membrane dynamics during its release. These findings collectively demonstrate that the incorporation of chromium into the hydroxyapatite lattice, as well as the presence of a dextran matrix, does not affect the cell’s viability. Thus, both 10CrHAp and 10CrHAp-Dx coatings could be considered safe candidates for further in vivo evaluation as bone tissue engineering or drug delivery scaffolds.

**Figure 19:**
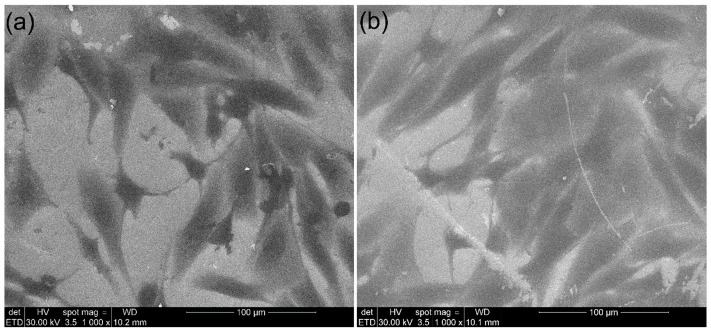
SEM micrographs of MG63 cells attachment on the surface of 10CrHAp (**a**) and 10CrHAp-Dx (**b**) coatings after 48 h incubation.

Complementary observations of MG63 osteosarcoma cell adhesion and morphological development on the surfaces of 10CrHAp and 10CrHAp-Dx coatings were obtained through scanning electron microscopy (SEM). After 48 h of incubation, the adhered cells were carefully fixed using glutaraldehyde to preserve structural details, dehydrated stepwise in ethanol, and finally air-dried prior to SEM examination. The resulting micrographs are presented in Figure 19.

Studying how MG63 cells interact with these coatings is essential for evaluating their potential use in biomedical applications, particularly for bone implants and graft materials. SEM is especially valuable in this context because it allows for the visualization of the cells in fine detail, capturing not only their attachment to the surface but also their spreading behavior, cytoplasmic extensions, and overall morphological state. These features provide important information about the health and functional response of cells in contact with the material. Examining the MG63 cells’ interactions with the surface of these coatings is important for assessing their suitability in biomedical applications, particularly for bone implants and grafts. SEM analysis has an important role as it offers a high-resolution visualization of cellular behavior, including surface attachment, spreading, cytoplasmic extensions, and overall morphology. These observations facilitate valuable insights into the health and functional response of cells in contact with the material. The SEM images presented in Figure 19a revealed that MG63 cells cultured on 10CrHAp coatings adhered successfully and maintained close contact with the substrate. More than that, the SEM micrographs highlighted that the cells displayed a relatively flattened and polygonal morphology, characteristic of osteoblast-like cells adapting to a surface. Furthermore, the SEM images revealed that the attached cells presented numerous filopodia extended from the cell edges, anchoring to the coating surface and suggesting active surface exploration. Some lamellipodia, which appear as broader sheet-like extensions, were also visible, contributing to the spreading process. These structures are important indicators of healthy adhesion, as they are involved in both stabilizing the cell on the substrate and initiating intracellular signaling pathways associated with proliferation and differentiation. In comparison, the SEM micrographs of cells on 10CrHAp-Dx coatings (Figure 19b) revealed even more extensive attachment and spreading. The cells on this surface appeared to be larger, more elongated, and showed a much greater degree of cytoplasmic expansion. The SEM micrographs emphasized the presence of broad lamellipodia spread widely across the surface, while an abundance of fine filopodia created multiple anchorage points with the substrate and, in some cases, interconnections with neighboring cells. This dense network of protrusions suggests that cells were not only firmly attached but also actively communicating with each other, forming the beginnings of a more integrated cell layer. Such behavior could indicate a more favorable microenvironment, one that could promote adhesion and signaling, and possibly exhibit osteogenic activity. The findings of the SEM observations showed that, on both coatings, the MG63 cells exhibited a healthy and flattened morphology, confirming successful adaptation to the surfaces. However, the results emphasized that the MG63 cells on 10CrHAp-Dx demonstrated more extensive spreading, greater numbers of adhesion-related structures, and stronger surface anchorage compared to those adhered to the surface of 10CrHAp coatings. These differences may be attributed to the presence of the dextran matrix, which likely contributes to the enhancement of the surface hydrophilicity and provides additional biochemical cues that facilitate the integrin binding and also the cytoskeletal organization. Taken together, these observations indicate that both the 10CrHAp and 10CrHAp-Dx coatings support the adhesion of MG63 cells, but the 10CrHAp-Dx surface offers a significantly more favorable environment for cellular spreading and anchorage. The SEM results therefore highlight the advantage of combining chromium-doped apatite with dextran matrix to optimize their biological performance. This finding, in combination with the complementary MTT assay results, strongly supports the potential application of these coatings in implant surface modification and bone tissue regeneration. The SEM findings are in agreement with the literature and align with numerous reports that highlight that organic–inorganic composites promote superior MG63 adhesion compared to pure hydroxyapatite. For example, Chen et al. [66], in their study, report that chitosan–silk sericin/HA nanocomposites support fusiform/polygonal morphologies with extensive pseudopodia anchorage and intercellular contacts by day 3. In the present study, the data showed that 10CrHAp-Dx already exhibited comparable spreading behaviors at 48 h, suggesting a more rapid onset of favorable adhesion. On the other hand, Sohn et al., in their study [67], reported that thin HA coatings on anodized Ti showed thickness-dependent effects on MG63 spreading and cytoplasmic extension, with thicker/rougher coatings providing stronger anchorage, therefore emphasizing that surface roughness and topography are also well-established regulators of osteoblast-like adhesion. Similarly, findings were published by Pan et al. [68] in which they showed that the MG63 seeded on micro/nano-hierarchical TiO_2_ coatings adhered more rapidly and spread earlier than on smooth Ti surfaces. These findings align well with our results and also suggest that the presence of dextran matrix in 10CrHAp-Dx may introduce nano-scale surface cues and enhanced hydrophilicity, which could help improve cell adhesion. Finally, studies conducted by Yoshida et al. and Tsai et al. regarding HA/collagen-based 3D scaffolds also report well-spread morphologies with actin stress fibers and intercellular networks [69,70]. While coatings cannot be compared to 3D scaffolds, the morphological features observed in our study through SEM and MM, like cell flattening, cytoplasmic extensions, and intercellular contacts, are qualitatively consistent with biomimetic organic–inorganic hybrids that support osteoblastic function.

**Figure 20:**
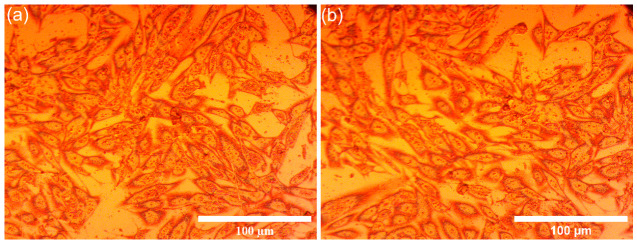
The morphology of MG63 cells incubated with 10CrHAp coatings (**a**) and 10CrHAp-Dx coatings (**b**) visualized by metallographic microscopy (20×) after 48 h of incubation.

Additional information regarding the MG63 cell adhesion and development on the 10CrHAp and 10CrHAp-Dx coatings after 48 h of incubation were obtained through metallographic microscopy analysis. This imaging technique provided a detailed evaluation of cell–surface interactions, particularly focusing on how effectively the coatings supported adhesion and subsequent cellular development. The microscopy observations, illustrated in Figure 20, demonstrated that both 10CrHAp and 10CrHAp-Dx coatings provided a suitable substrate for MG63 cell attachment and growth.

The MM visual analysis emphasized that the adhered cells displayed a typical MG63 morphology, having elongated and well-spread structures. Also, the MM images showed that no detectable morphological abnormalities were observed. This normal cellular appearance strongly suggests that the coatings do not induce cytotoxic effects and, more than that, they could create a favorable environment for cell anchorage and proliferation. These findings are in agreement with previous reports [71,72,73], which reported an enhanced MG63 cell viability with extended incubation times (24–48 h) on Cr-based hydroxyapatite coatings. The observed increase in cell viability over time highlights not only the biocompatibility of the coatings but also the bioactive nature of the 10CrHAp and 10CrHAp-Dx surfaces. The results highlight their ability to sustain essential cellular processes, including attachment, proliferation, and the early stages of differentiation. Although the precise molecular mechanisms governing the cell–coating interactions remain to be fully elucidated, the present study provides clear evidence of a positive biological response. The favorable adhesion and development of MG63 cells on both 10CrHAp and 10CrHAp-Dx coatings strongly support their potential use for biomedical applications, particularly in the design of bone-contact implants where both structural stability and biointegration are critical.

The AFM analysis of the coatings revealed a notable difference in surface roughness between the 10CrHAp and 10CrHAp-Dx coatings, with root-mean-square roughness (R_RMS_) values of 16.64 nm and 23.18 nm, respectively. This topographical modification has significant implications for the biological performance of the coatings, particularly in the context of osteoblast adhesion and early-stage osseointegration. Surface roughness at the nano-scale is widely recognized as a critical determinant of cellular response on biomaterial surfaces. Deligianni et al. [74] demonstrated that human bone marrow cells exhibited significantly enhanced adhesion and proliferation on rougher hydroxyapatite surfaces, establishing a positive correlation between surface roughness and osteoblast attachment. In their study, Price et al. [75] reported that nanometer-scale surface roughness selectively promoted osteoblast adhesion on carbon nanofiber compacts, while simultaneously reducing fibroblast attachment, suggesting that nano-scale topography can direct cell-selective responses favorable for bone tissue engineering. Lim et al. [76] investigated the regulation of integrin-mediated osteoblast focal adhesion on surfaces with nanopit depths ranging from 10 to 50 nm and found that nanotopographic features within this range significantly influenced focal adhesion kinase (FAK) signaling, integrin clustering, and cytoskeletal reorganization, with all of these parameters being essential for robust cell anchorage. Furthermore, Khang et al. [77] showed that nano-rough, micron-patterned titanium substrates enhanced osteoblast adhesion and alignment compared to smooth or solely micro-rough surfaces, reinforcing the importance of nano-scale surface features in directing osteoblast behavior. Additionally, the work by Deng et al. [78] confirmed that increased roughness on nanostructured biomaterial surfaces facilitated adhesion of both biomacromolecules and osteoblast cells, further supporting the positive trend observed in the present AFM data. It should be noted, however, that surface roughness alone is not fully responsible for cell response. Properties such as surface chemistry, wettability, hierarchical topography, and substrate stiffness collectively influence the cellular microenvironment. Therefore, the AFM findings in this study are encouraging and align well with reported literature studies.

**Figure 21:**
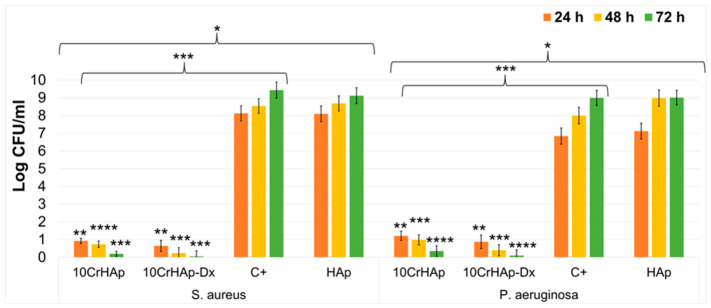
Graphical representation of the log colony forming units (CFUs)/mL of the HAp, 10CrHAp and 10CrHAp-Dx coatings incubated with Gram-negative *Pseudomonas aeruginosa* 27853 ATCC and Gram-positive *Staphylococcus aureus* ATCC 25923, for 24, 48, and 72 h. The results were represented as the mean values ± standard deviation (mean ± SD). Statistical analysis was performed using one-way ANOVA followed by post hoc Student’s *t*-test. Significance levels are indicated as follows: * *p* = 0.0001; ** *p* < 0.00000003; *** *p* < 0.00005; **** *p* < 0.0000001; and *p* ≥ 0.05 were considered non-significant.

The antibacterial activity of HAp, 10CrHAp and 10CrHAp-Dx coatings was investigated in vitro against two clinically relevant bacterial strains. The first, *Pseudomonas aeruginosa*, a common Gram-negative bacterium associated with severe bloodstream, pulmonary, and multi-organ infections. The second, *Staphylococcus aureus* ATCC 25923, a Gram-positive opportunistic pathogen frequently present in the upper respiratory tract. The antibacterial effects of both coatings were evaluated at three distinct time intervals to monitor bacterial growth inhibition over time. The results, expressed as the mean ± SD, are depicted graphically in Figure 21, demonstrating the coatings’ differential activity against the tested strains.

The in vitro antibacterial assays demonstrated a significant reduction in the colony-forming units (CFUs) of *Pseudomonas aeruginosa* after 24, 48, and 72 h of exposure to the 10CrHAp and 10CrHAp-Dx coatings. The data also showed that the HAp coatings did not inhibit the bacterial development. More than that, the in vitro antibacterial assays emphasized that the HAp coatings promote the *P. aeruginosa* and *S. aureus* bacterial proliferation and development on its surface. More than that, the results showed that the HAp coatings help promote the bacterial cells development and that the CFU incased with the incubation time. These findings are in good agreement with previously reported data demonstrating that hydroxyapatite (HAp) coatings can aid the development and colonization of microbial cells [79,80,81,82]. In their study, “Bacterial biofilm development on hydroxyapatite-coated glass”, Elliott et al. [79] showed that HAp-coated surfaces serve as favorable substrata for bacterial biofilm development, and surfaces’ cell attachment compared to uncoated surfaces. This could be attributed to the surface roughness and chemical composition of HAp, which provide favorable conditions for initial bacterial adhesion and subsequent biofilm maturation. Similar results were obtained in studies regarding biofilm formation on various dental and orthopedic materials that have confirmed that HAp-based surfaces tend to accumulate greater biofilm mass relative to other ceramic or metallic substrates [79,80,81,82,83]. The study reported by Iconaru et al. [81], “Investigation of Spin Coating Cerium-Doped Hydroxyapatite Thin Films with Antifungal Properties”, demonstrated that undoped HAp coatings supported the adherence and proliferation of *Candida albicans*, confirming that HAp surfaces can facilitate fungal cell development. Furthermore, Gottardo et al. [82] reported that silver-loaded HAp nanocomposites were specifically designed to counteract the inherent susceptibility of HAp to fungal colonization, further underscoring the biocompatibility of HAp with microbial organisms.

The quantitative analysis indicated that the antibacterial effect of the 10CrHAp-Dx coating was more pronounced, resulting in a greater decrease in CFUs compared to both the control and the 10CrHAp coating. Moreover, the assays revealed that the antibacterial activity was influenced by the incubation time, showing a stronger reduction in CFUs for both coatings as the incubation duration increased. This time-dependent effect can be attributed to the gradual and sustained release of chromium ions, which are known to disrupt bacterial membranes and interfere with metabolic processes, thereby inhibiting cell growth [84,85]. In recent years, *Pseudomonas aeruginosa* has drawn increasing attention due to its role as a leading cause of healthcare-associated infections and its remarkable intrinsic and acquired resistance to multiple antibiotics. The resistance mechanisms reported to be exhibited by *Pseudomonas aeruginosa* are complex and contribute greatly to its persistence in clinical settings. The intrinsic mechanisms include low outer membrane permeability, which restricts the entry of many antimicrobial agents, and the overexpression of efflux pumps that actively expel a broad spectrum of antibiotics. Additionally, the production of β-lactamases, particularly AmpC, provides enzymatic degradation of β-lactam compounds, further diminishing therapeutic efficacy. In the case of *P. aeruginosa*, the biofilm formation represents another crucial problem in the case of infections due to the fact that biofilm-embedded cells display a noticeably reduced susceptibility to antibiotics compared to their planktonic counterparts, largely due to limited drug penetration and altered metabolic activity. In addition to these intrinsic properties, *P. aeruginosa* also demonstrated a remarkable capacity to acquire resistance through horizontal gene transfer. This feature helps the accumulation of resistance genes that confer protection against multiple antibiotic classes, including carbapenems, fluoroquinolones, and aminoglycosides. The combined effect of these intrinsic and acquired mechanisms results in a multidrug-resistant phenotype that severely complicates clinical management. Therefore, the clinical implications of this multidrug resistance are significant. This multidrug resistance complicates treatment strategies and contributes to high morbidity and mortality rates, particularly among immunocompromised and hospitalized patients. The resilience of *P. aeruginosa* against last-resort antibiotics underscores the urgent need for alternative therapeutic strategies, including combination therapies, novel antimicrobial agents, and approaches aimed at disrupting biofilm integrity or inhibiting efflux pump activity [86,87,88]. Moreover, the data suggested that the 10CrHAp-Dx coatings exhibited better antibacterial activity than 10CrHAp coatings against both *P. aeruginosa* and *S. aureus* strains. The superior antibacterial activity observed for the 10CrHAp-Dx coating may result from the synergistic interaction between chromium ion release and the dextran matrix. These findings could be attributed to the fact that, dextran, with its hydrophilic and bioactive properties, has the ability to enhance the ion diffusion as well as surface interactions with bacterial cells, thereby amplifying the inhibitory effect [89,90,91,92]. This interaction could explain the stronger inhibition of *P. aeruginosa* and *S. aureus* growth observed in the case of 10CrHAp-Dx compared to 10CrHAp coatings. The antibacterial assays demonstrated that the incorporation of dextran into 10CrHAp could enhance its antibacterial effects against both Gram-positive (*Staphylococcus aureus*) and Gram-negative (*Pseudomonas aeruginosa*) strains. The in vitro assays revealed that while 10CrHAp coatings exhibited significant inhibitory activity and managed to considerably reduce the values of the bacterial CFUs after 72 h of incubation, the 10CrHAp-Dx coating showed an even stronger effect, leading to an almost complete eradication of the viable colonies across all tested strains at the same time point. These findings could be attributed to a synergistic effect between 10CrHAp and dextran, with the polysaccharide matrix conferring an additional advantage in suppressing bacterial proliferation.

The antibacterial efficacy of the coatings was also evaluated against *P. aeruginosa* and *S. aureus*, with the results expressed as log reductions and corresponding percentage decreases in viable bacterial counts relative to the positive control (C+). In the case of *S. aureus*, after 24 h of incubation, the C+ showed approximately 8.1 log CFU/mL, while the incubation with 10CrHAp and 10CrHAp-Dx managed to reduce the counts to about 0.9 and 0.6 log CFU/mL. These values correspond to log reductions of approximately 7.2 and 7.5 or higher than 99.99% reduction in viable bacteria. In contrast, HAp coatings exhibited negligible antibacterial activity, maintaining the bacterial levels comparable to the control, thus indicating that chromium ions and dextran matrix are responsible for the observed antibacterial effect. After 48 h, the bacterial counts obtained for the control medium (C+) and HAp coatings increased to approximately 8.5–8.6 log CFU/mL, while 10CrHAp and 10CrHAp-Dx reduced the bacterial counts to approximately 0.7 and 0.25 log CFU/mL, corresponding to log reductions of approximately 7.8 and 8.3, demonstrating enhanced antibacterial activity over time. After 72 h, the difference became even more pronounced, with the control reaching approximately 9.4 log CFU/mL, while 10CrHAp and 10CrHAp-Dx decreased the bacterial counts to approximately 0.2 and nearly zero (~0.05) log CFU/mL, leading to log reductions of approximately 9.2 and ~9.35 and a kill rate exceeding 99.99%. These results highlight that continuous decline in bacterial counts over 24–72 h indicate a sustained bactericidal effect rather than a merely bacteriostatic action. In the case of *P. aeruginosa*, which is a Gram-negative bacterium known to be more resistant to antimicrobial agents due to its protective outer membrane, the antibacterial assays also exhibited significant susceptibility to both 10CrHAp and 10CrHAp-Dx coatings. After 24 h, the positive control (C+) showed approximately 6.8 log CFU/mL, while the incubation with 10CrHAp and 10CrHAp-Dx helped to reduce the bacterial counts to about 1.2 and 0.9 log CFU/mL, corresponding to log reductions of approximately 5.6 and ~5.9, or greater than 99.99% reduction in viable cells. On the other hand, HAp coatings demonstrated no significant antibacterial activity, with counts remaining around 7.1 log CFU/mL. After 48 h, the bacterial growth in the control increased to approximately 8.0 log CFU/mL, while the incubation with 10CrHAp and 10CrHAp-Dx led to decreased counts of approximately 1.0 and 0.35 log CFU/mL, leading to log reductions of approximately 7.0 and ~7.65 (>99.99% reduction). After 72 h, the control reached a value of about 9.0 log CFU/mL, while the incubation with 10CrHAp and 10CrHAp-Dx coatings lead to the further decline to approximately 0.4 log CFU/mL for 10CrHAp and approximately 0.1 log CFU/mL for 10CrHAp-Dx, corresponding to log reductions of ~8.6 and ~8.9 and exceeding 99.99% bacterial elimination, confirming a strong and sustained antibacterial effect over time. The antibacterial assays highlighted that across both bacterial strains and for all tested time intervals 10CrHAp-Dx coatings exhibited a greater antibacterial activity compared to 10CrHAp by an additional 0.3–0.5 log reduction. More than that, the results suggested that a clear time-dependent bactericidal effect can be observed for both 10CrHAp and 10CrHAp-Dx coatings. These results could be attributed to a sustained Cr^3+^ ion release from the hydroxyapatite matrix, supporting the potential of these coatings to be used in biomedical applications with prolonged antibacterial properties.

**Figure 22:**
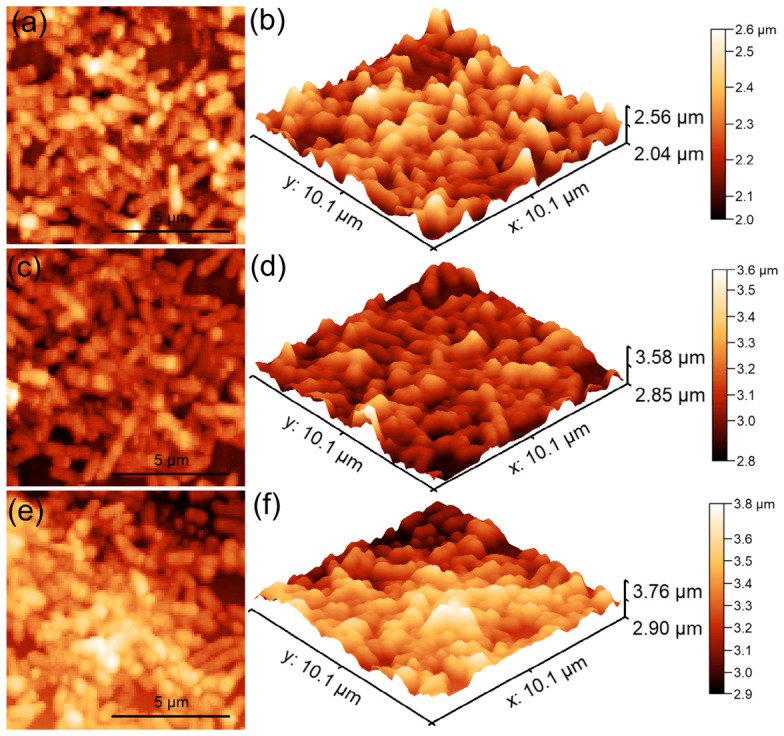
Two-dimensional AFM topography images of *Pseudomonas aeruginosa* ATCC 27853 cells adhered to the surface of HAp coatings after 24 (**a**), 48 (**c**), and 72 h (**e**) of incubation, along with their corresponding 3D representations (**b**,**d**,**f**).

**Figure 23:**
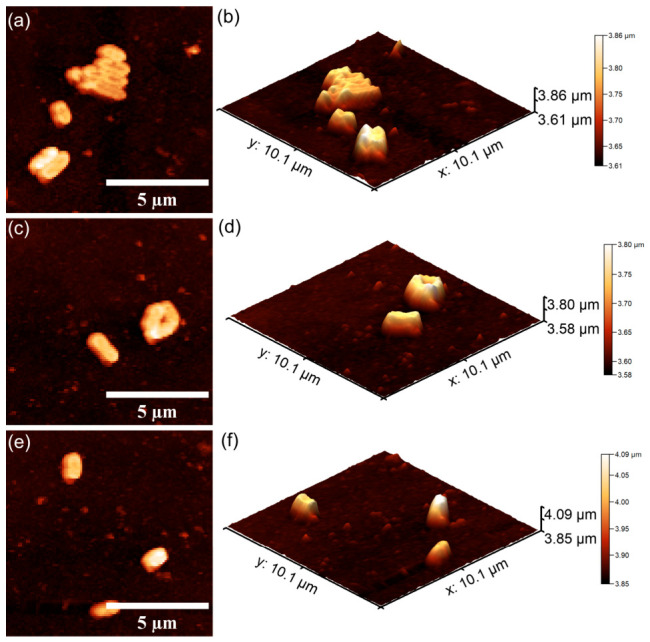
Two-dimensional AFM topography images of *Pseudomonas aeruginosa* ATCC 27853 cells adhered to the surface of 10CrHAp coatings after 24 (**a**), 48 (**c**), and 72 h (**e**) of incubation, along with their corresponding 3D representations (**b**,**d**,**f**).

**Figure 24:**
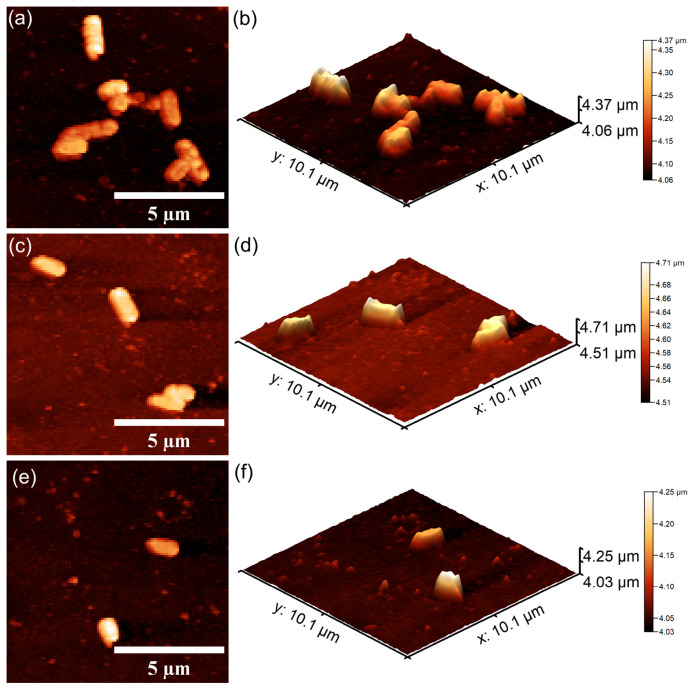
Two-dimensional AFM topography images of *Pseudomonas aeruginosa* ATCC 27853 cells adhered to the surface of 10CrHAp-Dx coatings after 24 (**a**), 48 (**c**), and 72 h (**e**) of incubation, along with their corresponding 3D representations (**b**,**d**,**f**).

In addition, atomic force microscopy (AFM) investigations were carried out to evaluate the adhesion behavior and proliferation patterns of both *P. aeruginosa* and *S. aureus* bacterial cells on the surfaces of HAp, 10CrHAp and 10CrHAp-Dx coatings. These studies aimed to provide a deeper understanding of the antibacterial contributions of chromium ions and dextran matrix. For this purpose, the coatings were incubated with *P. aeruginosa* and *S. aureus* bacterial suspensions for three different time intervals, 24, 48, and 72 h. The incubation was performed under ambient conditions and at room temperature. After each incubation period, AFM topographies were recorded on the surface of the coatings in order to investigate the surface morphological changes associated with bacterial attachment and colonization. Two-dimensional (2D) topographical scans were obtained in non-contact mode over defined surface areas of 10.1 × 10.1 µm^2^, providing high-resolution insights. In addition to the 2D AFM topographies, corresponding three-dimensional (3D) reconstructions were generated to further visualize the spatial distribution of the bacterial cells and the surface modifications. The comparative AFM images for the attachment and proliferation of *P. aeruginosa* on the surface of HAp,10CrHAp and 10CrHAp-Dx coatings are depicted in Figure 22, Figure 23 and Figure 24, highlighting the differences in bacterial interactions and coating surface responses over time.

AFM investigations revealed that both 10CrHAp and 10CrHAp-Dx coatings effectively inhibited the initial adhesion and the development of *P. aeruginosa* cells, while the HAp coating promote the *P. aeruginosa* bacterial strain development. The AFM topography of the surface of HAp coatings incubated for 24 h with *P. aeruginosa* reveals the presence of an early-stage biofilm development which is characterized by the adhesion of individual *P. aeruginosa* cells followed by small microcolonies formation on the HAp surface. The 2D AFM images depict the presence of scattered rod-shaped bacterial cells, which could be observed as both individually as well as in small clusters. The images highlight that the HAp coating does not appear to inhibit the initial *P. aeruginosa* bacterial adhesion. These findings are in agreement with previous reported studies that unmodified hydroxyapatite lacks intrinsic bactericidal activity against Gram-negative organisms [93,94,95].

After 48 h, the AFM images depict a significant increase in bacterial cell surface coverage. The biofilm now presents a denser, more confluent coverage with distinct three-dimensional features. This stage could be attributed to active biofilm maturation, where *P. aeruginosa* colonies have exhibit a rapid proliferation rate. At 72 h, the AFM topography reveals a fully mature, multilayered biofilm exhibiting a complex architecture typical of *P. aeruginosa* biofilms. These findings underscore that while HAp coatings alone do not prevent *P. aeruginosa* biofilm maturation, the resultant biofilm reaches a structurally resilient state in the first 72 h, thus highlighting the need for the surface functionalization of HAp surface to be able to help prevent biofilm-associated infection risks on implant surfaces. Furthermore, the AFM topographical images (Figure 22a–f) reveal the clear time-dependent progression of *P. aeruginosa* biofilm development on hydroxyapatite (HAp) coatings over 24, 48, and 72 h of incubation.

The AFM analysis revealed that the 10CrHAp and 10CrHAp-Dx coatings not only prevent early attachment of bacterial cells but also succeed in inhibiting the biofilm formation on the coating’s surfaces.

The 2D topographies showed that the adhered bacterial cells retained their characteristic rod-shaped morphology, with dimensions ranging from 1.18 to 2.75 µm in length and 0.53–0.85 µm in width. In addition, the AFM studies revealed that 10CrHAp-Dx demonstrated superior antibacterial activity compared to 10CrHAp, suggesting a synergistic effect between chromium ion release and the functional properties of dextran. A pronounced reduction in bacterial attachment was observed within the first 24 h of incubation, as shown by both 2D and 3D AFM images. This inhibitory effect increased with the increase in the incubation time. After 72 h of incubation, only a few isolated *P. aeruginosa* cells were detectable on the surface of either coating. These findings confirm that the coatings efficiently prevented bacterial colonization and biofilm maturation over prolonged periods.

**Figure 25:**
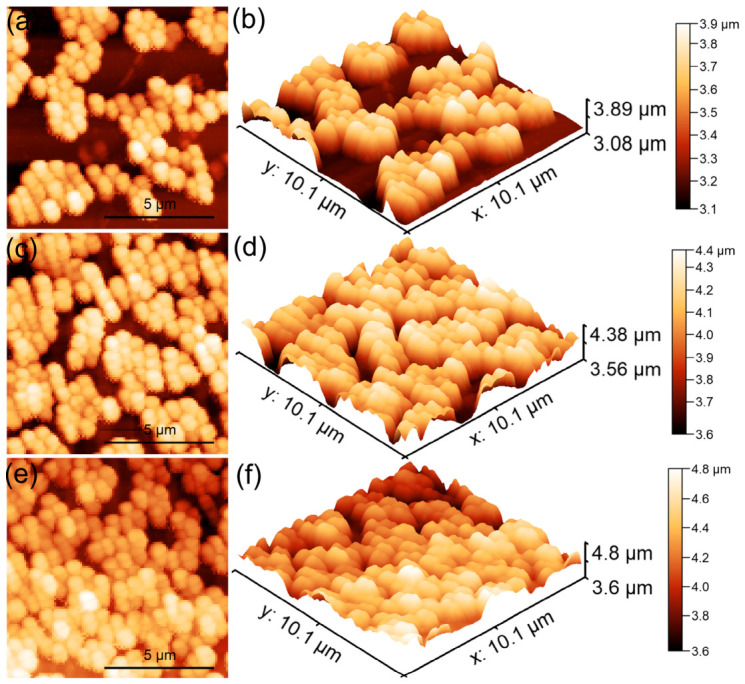
Two-dimensional AFM topography images of *Staphylococcus aureus* ATCC 25923 cells adhered to the surface of HAp coatings after 24 (**a**), 48 (**c**), and 72 h (**e**) of incubation, along with their corresponding 3D representations (**b**,**d**,**f**).

**Figure 26:**
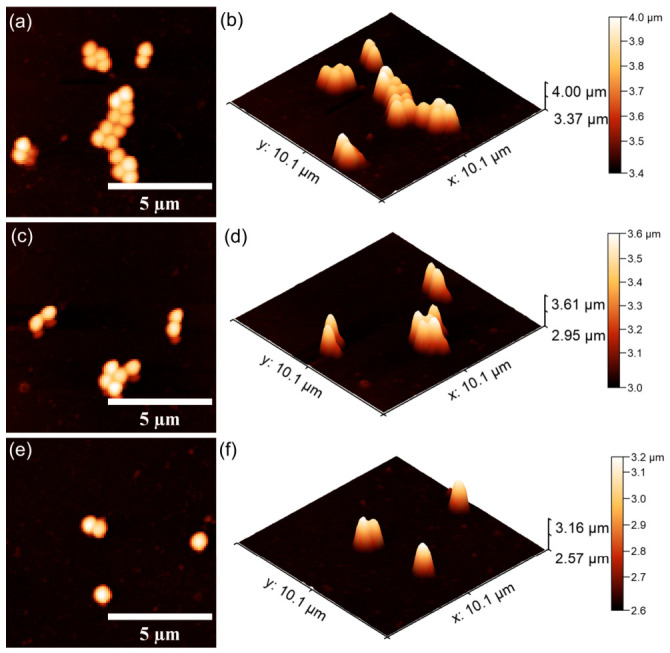
Two-dimensional AFM topography images of *Staphylococcus aureus* ATCC 25923 cells adhered to the surface of 10CrHAp coatings after 24 (**a**), 48 (**c**), and 72 h (**e**) of incubation, along with their corresponding 3D representations (**b**,**d**,**f**).

**Figure 27:**
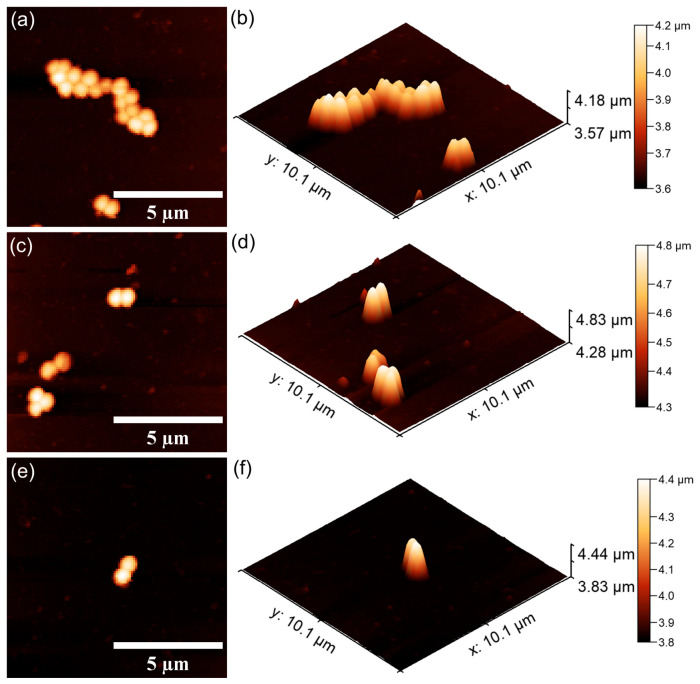
Two-dimensional AFM topography images of *Staphylococcus aureus* ATCC 25923 cells adhered to the surface of 10CrHAp-Dx coatings after 24 (**a**), 48 (**c**), and 72 h (**e**) of incubation, along with their corresponding 3D representations (**b**,**d**,**f**).

The AFM topographies of *S. aureus* cells adhered to the HAp, 10CrHAp and 10CrHAp-Dx coatings are shown in Figure 25, Figure 26 and Figure 27. Both the 2D AFM topographies as well as their 3D representations demonstrated that 10CrHAp and 10CrHAp-Dx coatings effectively suppressed *S. aureus* biofilm formation on their surfaces, while the HAp coating promoted the *S. aureus* bacterial strain development. The AFM topographies depicting the development of attachment of S aureus on the surface of HAp coatings after 24 h of incubation (Figure 25a,b) show that individual bacterial cells and small clusters are clearly distinguishable on the HAp surface. The characteristic spherical morphology of *S. aureus* with dimensions of approximately 0.5–1 µm in diameter is well observed, with cells appearing as discrete, rounded features. After 48 h of incubation the AFM topographies, as depicted in Figure 25c,d, show that a significant increase in bacterial surface coverage is observed. It can be observed that the bacterial clusters have expanded and begun forming a more continuous biofilm layer across the HAp surface. The AFM topographies after 72 h (Figure 25e,f) highlight that the bacterial biofilm has matured significantly. The 2D image shows a near-complete surface coverage with densely packed bacterial aggregates. These findings demonstrate that the HAp surface is susceptible to *S. aureus* colonization and that also supports the development and maturation of bacterial biofilm over 72 h.

Furthermore, the AFM studies showed that the 10CrHAp and 10CrHAp-Dx coatings exhibited a strong inhibitory effect during the early adhesion phase of the *S. aureus* bacterial cells within the first 24 h. The 2D AFM images provided additional confirmation that the *S. aureus* cells adhered to the coating surfaces preserved their characteristic spherical morphology, a defining feature of this bacterial species, with diameters ranging from 0.52 to 0.93 µm [96]. This observation suggests that while adhesion occurred, the coatings effectively limited subsequent cell proliferation and aggregation. Furthermore, the AFM analysis revealed a time-dependent relation of the antibacterial activity of the coatings, showing that the inhibitory activity of both coatings became more pronounced with the increase in the incubation period, thereby reducing the extent of biofilm development over time.

The AFM studies demonstrated that the decrease in the number of *S. aureus* cells adhered to the coating surfaces was influenced by the incubation time. More than that, the data showed that the bacterial colonization was significantly reduced after 72 h, where only a few dispersed cells could be observed on both 10CrHAp and 10CrHAp-Dx coatings. This progressive reduction, which was highlighted by both 2D AFM topographies as well as their 3D reconstruction, emphasizes the coatings’ ability to interfere with the bacterial adhesion and colonization. The anti-adhesive effect that was observed can be attributed to the physicochemical modifications introduced by chromium doping and the dextran matrix, which are likely to alter the surface parameters of the coatings such as the roughness, hydrophilicity, surface charge distribution, and chemical functionality. These factors are known to play critical roles in modulating bacterial adhesion by influencing electrostatic interactions, van der Waals forces, and hydrogen bonding at the biomaterial–bacteria interface [97,98]. Considering the well-recognized ability of *S. aureus* to colonize biomaterial surfaces through adhesin-mediated interactions and to progress toward mature biofilms via polysaccharide intercellular adhesin (PIA) and extracellular polymeric substances (EPS), the pronounced reduction in surface-associated bacterial cells observed in the case of both 10CrHAp and 10CrHAp-Dx coatings could indicate that their surfaces interfere not only with the early adhesion phase but also with the irreversible attachment and also biofilm development. This inhibitory effect has a significant clinical relevance, due to the fact that *S. aureus* biofilms are notoriously linked to persistent device-related infections, the evasion of host immune defenses, and a heightened tolerance to conventional antimicrobial therapies [96]. The antibacterial performance of these coatings is particularly advantageous for biomedical applications where implantable devices like bone grafts, prosthetics, and dental implants are sensible to the microbial colonization and development of persistent biofilm. Moreover, their ability to limit bacterial adhesion and biofilm development suggests potential utility in wound care applications, where infection prevention is critical for effective healing. The enhanced antibacterial properties of 10CrHAp-Dx, in particular, may arise from complementary mechanisms: chromium ions interfere with bacterial metabolic activity and membrane integrity, while dextran contributes to altered surface physicochemistry and the inhibition of biofilm matrix formation. Together, these cooperative effects underscore the potential of 10CrHAp-Dx as a multifunctional antibacterial coating for advanced biomedical use. The primary reported antibacterial mechanism of action of chromium ions is attributed to their ability to disrupt the bacterial membrane integrity, thus causing the loss of some essential intracellular components like ions, proteins, and nucleotides, ultimately conducting to cell lysis [73,99]. Cr^3+^ ions usually interact electrostatically with the negatively charged components of the bacterial outer membrane, in particular with the lipopolysaccharides, and compromise the membrane structure, increasing permeability, and inducing leakage of the cellular contents [73,99]. In addition to membrane disruption, chromium ions exhibit antibacterial effects by promoting the generation of reactive oxygen species (ROS), which have the ability to induce oxidative damage to critical cellular structures, including DNA, proteins, and lipids. This oxidative stress can lead to some genetic mutations, protein denaturation, and lipid peroxidation, further altering the cell’s viability. While membrane disruption and ROS-mediated damage are well-established mechanisms, the complete spectrum of Cr^3+^ antibacterial activity remains yet to be fully understood. Current research continues to explore how these ions interact with bacterial cells at the molecular level, including potential effects on metabolic pathways, enzyme function, and signal transduction. A deeper understanding of these interactions could inform the design of more effective chromium-based antimicrobial strategies [73,99,100,101]. The findings also suggested that the presence of a dextran matrix significantly enhanced the antibacterial performance of chromium-doped hydroxyapatite coatings. The 10CrHAp coatings exhibited partial antibacterial activity, which can be attributed to the release of Cr ions and their ability to disrupt bacterial metabolism. However, the presence of the dextran matrix further amplified this effect, indicating a synergistic mechanism. Therefore, the improved activity can be explained by several factors. First, dextran prevented the agglomeration of CrHAp particles, ensuring better dispersion and a larger effective surface area. This could have facilitated a more controlled and sustained release of Cr ions into the surrounding medium, thereby prolonging the antibacterial effects. Second, dextran, being a hydrophilic polysaccharide, has the ability to promote closer interactions between the nanocomposite and bacterial cell walls. This likely could have led to an enhancement in the local concentration of Cr ions at the bacterial surface, leading to increased membrane damage. These findings demonstrate that 10CrHAp and 10CrHAp-Dx coatings present surface physicochemical properties that have the ability to effectively inhibit bacterial cells’ adhesion and biofilm formation, thus making them strong candidates for the development of future bioactive, infection-resistant biomedical devices.

Doped and undoped HAp-based coatings, as well as layers of HAp embedded in various polymer matrices, such as PVA or chitosan, have been widely investigated due to their ability to improve physico-chemical and biological properties, including improved cell viability and adhesion to metal or Si substrates [102,103,104,105]. Similarly, Cr-doped hydroxyapatite incorporated into a PVA matrix has been reported to exhibit good structural homogeneity and biocompatibility, confirming the potential suitability of polymer-based materials for biomedical coatings [33]. In comparison, dextran-based matrices offer additional advantages due to their high hydrophilicity, strong biocompatibility, and ability to stabilize hydroxyapatite suspensions while promoting uniform film formation and good substrate adhesion. Recent studies on dextran–hydroxyapatite composites have demonstrated smooth, homogeneous coatings and good cell compatibility, further supporting their suitability for biomedical applications [32].

Therefore, the results obtained in this study suggest that the 10CrHAp and 10CrHAp-Dx coatings offers comparable or improved coating properties compared to chitosan- and PVA-based HAp coatings, while highlighting the contribution of dextran as a matrix that improves overall coatings properties. All these findings support the novelty of using dextran as an alternative to organic matrix for Cr-doped hydroxyapatite coatings.

## 4. Conclusions

This study reports, for the first time, on the preparation of 10CrHAp (x_Cr_ = 0.1) and 10CrHAp-Dx coatings via spin coating from gels obtained through an adapted sol–gel method. The XRD results and Rietveld refinement confirm that chromium incorporation and subsequent dextran loading induce controlled lattice expansion and moderate microstructural changes while maintaining the phase purity and structural integrity of hydroxyapatite. These findings validate Cr-doped HAp as a robust and responsive host matrix for dextran loading. The SEM analysis showed that the surface morphology consists of nanoparticle conglomerates while the MM results confirmed that the studied surfaces are uniform and continuous. The EDS results demonstrated the purity of the 10CrHAp and 10CrHAp-Dx coatings, and the FTIR analysis confirmed the presence of characteristic hydroxyapatite vibrational bands in both coatings. Moreover, the FTIR data underline the presence of functional groups of dextran in 10CrHAp-Dx. Overall, the spin coating technique enabled the formation of continuous coatings. The AFM investigation revealed that both 10CrHAp and 10CrHAp-Dx coatings exhibited a continuous and uniform surface with good surface stability. The 10CrHAp coating exhibited a smooth and homogeneous morphology with evenly distributed nanometric particle clusters, while the presence of dextran matrix in 10CrHAp-Dx further enhanced the surface uniformity and led to the formation of more pronounced, evenly distributed agglomerates. Quantitative roughness analysis showed an increase in R_RMS_ from 16.64 nm for 10CrHAp to 23.18 nm for 10CrHAp-Dx, indicating that dextran promotes a slightly rougher and more textured surface. This controlled increase in roughness, together with the improved homogeneity, suggests that the 10CrHAp-Dx coating may offer superior interfacial properties and greater potential for biomedical applications, particularly where enhanced cell adhesion and biological integration are needed. The in vitro biocompatibility assessment demonstrated that both 10CrHAp and 10CrHAp-Dx coatings possess favorable biological properties. The results of the MTT assay confirmed a high proportion of viable MG63 cells, indicating excellent cytocompatibility. Furthermore, the complementary SEM and MM observations also showed that the cells adhered effectively to the coating’s surfaces and maintained a typical osteoblast-like morphology. Collectively, these findings suggest that the coatings can support cell adhesion and proliferation without inducing cytotoxic effects, thereby confirming their potential as promising candidates for bone tissue engineering and implant-related applications.

The in vitro antibacterial properties of the 10CrHAp and 10CrHAp-Dx coatings against *P. aeruginosa* and *S. aureus* was assessed using in vitro antibacterial assays, which confirmed their strong inhibitory capacity. The results showed that both coatings demonstrated a pronounced antibacterial activity. Furthermore, the findings suggested that 10CrHAp-Dx exhibited a significantly greater inhibitory effect compared to 10CrHAp, thus highlighting the role of dextran in possibly enhancing the antibacterial efficacy of the 10CrHAp coating. This improvement may be attributed to the modifications of the surface physicochemical characteristics as well as potential alterations in the chemical composition and ion release kinetics, induced by the presence of dextran matrix, all of which can synergistically contribute to bacterial membrane destabilization, interference with metabolic pathways, and suppression of biofilm initiation. Complementary AFM analyses further confirmed these findings by revealing marked alterations in the adhesion behavior of both *P. aeruginosa* and *S. aureus* bacterial cells on the surfaces of 10CrHAp and 10CrHAp-Dx coatings, thereby supporting the coatings’ ability to prevent early colonization. Collectively, these results highlight the antibacterial potential of 10CrHAp and 10CrHAp-Dx. These properties are particularly relevant to the development of infection-resistant biomedical coatings, as they provide a dual function of supporting regenerative applications while simultaneously decreasing the risk of device-associated infections. Consequently, 10CrHAp and 10CrHAp-Dx could be considered as promising candidates for next-generation biomaterial coatings in regenerative medicine and advanced medical device design. However, it is important to note that the potential chromium-related toxicity and the absence of in vivo validation are key limitations of this study, highlighting the need for further, more comprehensive investigations into biocompatibility and in vivo behavior.

## Figures and Tables

**Table 1:** The lattice parameters, average crystallite size and unit cell volume.

Sample	Lattice Parameter (Å)		c/a	Average Crystal Size (nm)	Unit Cell Volume (Å^3^)
	a-Axis	c-Axis			
10CrHAp	9.321	6.868	0.736	21.72	516.78
10CrHAp-Dx	9.371	6.876	0.728	20.46	522.87

**Table 2:** Lattice parameters and R-values for 10CrHAp and 10CrHAp-Dx samples obtained by Rietveld refinement.

Sample	Rwp (%)	Rp (%)	χ^2^ (%)	a (Å)	c (Å)	D (nm)	V (Å^3^)
JCPDS No. 00-009-0432	-	-	-	9.418	6.884	-	528.08
10CrHAp	6.321	4.903	0.225	9.423	6.889	23.36	529.730
10CrHAp-Dx	6.295	5.003	0.228	9.429	6.907	19.83	531.804

**Table 3:** Chemical composition of 10CrHAp and 10CrHAp-Dx samples obtained from XPS.

XPS-Chemical Composition			
10CrHAp		10CrHAp-Dx	
Chemical Element	At. %	Chemical Element	At. %
Ca	23.2	Ca	17.7
P	19.6	P	16.3
O	33.7	O	38.7
Cr	9.5	Cr	9.3
C	14	C	18

**Table 4:** Chemical composition of 10CrHAp and 10CrHAp-Dx samples obtained from EDX semi-quantitative analysis.

EDX-Chemical Composition			
10CrHAp		10CrHAp-Dx	
Chemical Element	At. %	Chemical Element	At. %
Ca	28.2	Ca	20.4
P	22.8	P	18.1
O	39.2	O	44.7
Cr	9.8	Cr	9.6
-	-	C	7.2

## Data Availability

The original contributions presented in the study are included in the article; further inquiries can be directed to the corresponding authors.
